# Supplementary material for: Identification of a Novel Tumor Microenvironment Prognostic Signature for Advanced-Stage Serous Ovarian Cancer
Source: Cancers (Basel). 2021 Jul 3;13(13):3343. doi: 10.3390/cancers13133343 (PMC8268985; doi:10.3390/cancers13133343)
Supplement: Supplementary file 1 [file cancers-13-03343-s001.zip › cancers-1262252-supplementary.pdf]

cancers

Article

Identification of a Novel Tumor Microenvironment Prognostic Signature for Advanced-Stage Serous Ovarian Cancer

Mingjun Zheng, Junyu Long, Anca Chelariu-Raicu, Heather Mullikin, Theresa Vilsmaier, Aurelia Vattai, Helene Hildegard Heidegger, Falk Batz, Simon Keckstein, Udo Jeschke, Fabian Trillsch, Sven Mahner and Till Kaltofen

Citation: Zheng, M.; Long, J.; Chelariu-Raicu, A.; Mullikin, H.; Vilsmaier, T.; Vattai, A.; Heidegger, H.H.; Batz, F.; Keckstein, S.; Jeschke, U.; et al. Identification of a Novel Tumor Microenvironment Prognostic Signature for Advanced-Stage Serous Ovarian Cancer. *Cancers* **2021**, *13*, 3343. https://doi.org/10.3390/cancers13133343

Academic Editor: Mary F McMullin

Received: 31 May 2021
Accepted: 29 June 2021
Published: 3 July 2021

Publisher's Note: MDPI stays neutral with regard to jurisdictional claims in published maps and institutional affiliations.

Copyright: © 2021 by the authors. Submitted for possible open access publication under the terms and con-

Supplementary Materials:

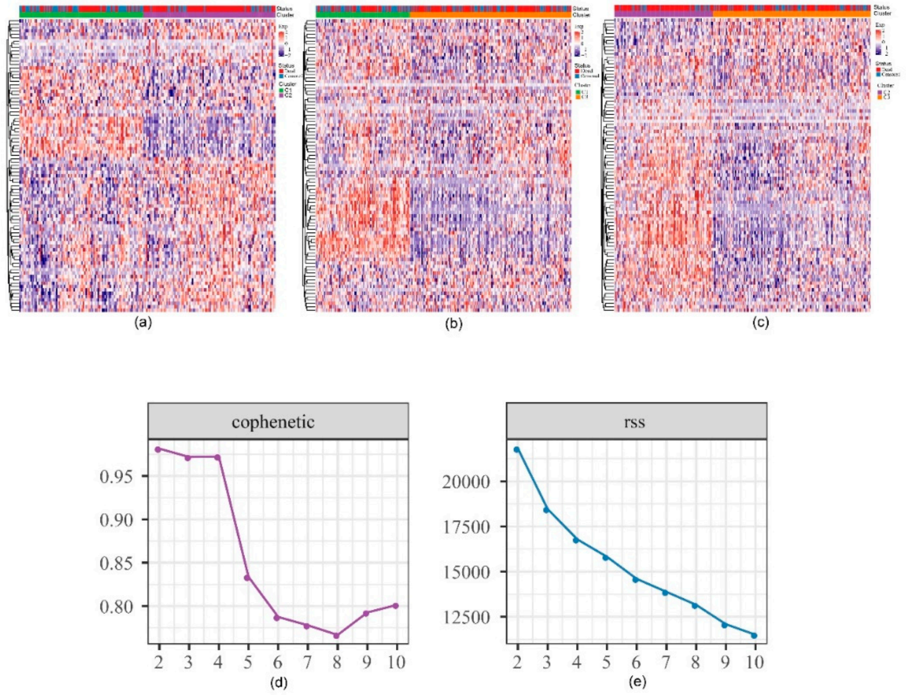

Figure S1. Expression of prognostic TME-related genes between the three clusters: (**a-c**) Heatmaps comparing C1, C2 and C3; (**d**) The cophenetic correlation coefficient is used to reflect the stability of the cluster obtained from NMF; (**e**) rss is used to reflect the clustering performance of the model.

ditions of the Creative Commons Attribution (CC BY) license (http://creativecommons.org/licenses/by/4.0/).

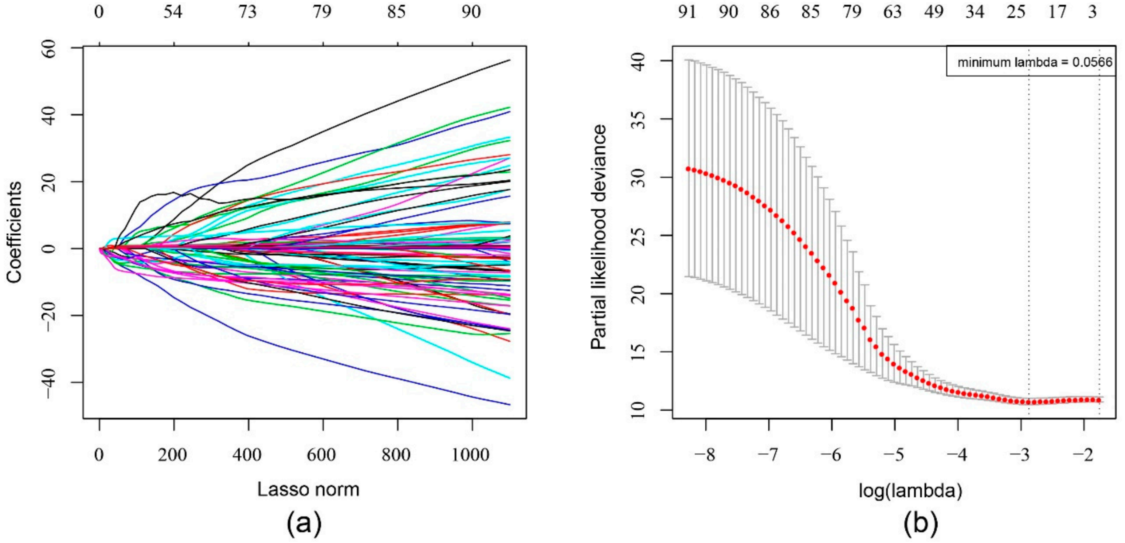

Figure S2. Analysis of lasso regression: (**a**) Changing trajectory of each independent variable (the abscissa represents the corrected lambda and the ordinate represents the coefficient of the independent variable); (**b**) log value of the independent variable lambda (the abscissa represents the CI of each lambda, and the ordinate represents errors in cross-validation).

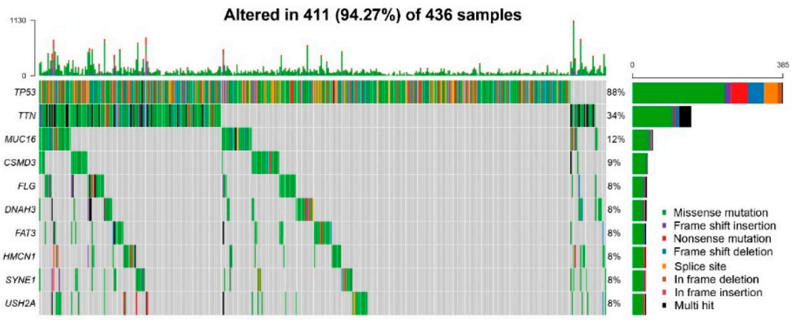

Figure S3. Distribution of common single nucleotide mutations in OC using TCGA biolinks package with 94.27% samples altered.

Table S1. 4061 transcriptome-specific TME-related genes.

**ZWINT**	**SRPK1**	**PPP1R2**	**MIEN1**	**HIST1H3J**	**DPP10**	**CASP3**
ZSCAN5B	SRMS	PPP1R16B	MICOS13	HIST1H3H	DPM3	CASKIN1
ZSCAN10	SRL	PPP1R16A	MICB	HIST1H3F	DPM1	CASD1
ZPBP2	SRGN	PPP1R14B	MICAL3	HIST1H3A	DPF1	CARD9
ZP3	SRD5A3	PPP1CC	MICAL2	HIST1H2BO	DPEP2	CARD8
ZNF99	SRCIN1	PPM1N	MIA	HIST1H2BN	DPCD	CARD19
ZNF92	SRC	PPM1L	MGST1	HIST1H2BL	DONSON	CARD11
ZNF91	SRA1	PPM1J	MGAT5B	HIST1H2BJ	DOK5	CAPZA1
ZNF90	SQSTM1	PPM1H	MGAM	HIST1H2BH	DOCK9	CAPN3
ZNF878	SPX	PPM1F	MFSD4B	HIST1H2BG	DOCK6	CAPN13
ZNF789	SPTSSB	PPM1E	MFSD3	HIST1H2BF	DOCK5	CAPN12
ZNF747	SPTAN1	PPM1B	MFSD2B	HIST1H2BC	DOCK2	CAPG
ZNF716	SPSB4	PPIB	MFSD10	HIST1H2BB	DOCK10	CAMP
ZNF710	SPRY3	PPIAL4G	MFGE8	HIST1H2AM	DOC2B	CAMLG
ZNF707	SPRY2	PPIAL4C	MFAP5	HIST1H2AL	DOC2A	CAMKV
ZNF703	SPP1	PPIA	MFAP4	HIST1H2AJ	DNTTIP1	CAMKK2

ZNF695	SPON2	PPFIBP2	MFAP3L	HIST1H2AG	DNMT3B	CAMK2N2
ZNF692	SPON1	PPFIBP1	MEX3A	HIST1H2AE	DNMT3A	CALR
ZNF681	SPOCK2	PPFIA4	METTL9	HIST1H2AD	DNMT1	CALN1
ZNF668	SPOCK1	PPFIA2	METTL27	HIST1H1D	DNM1	CALHM3
ZNF629	SPN	PPFIA1	METTL26	HIST1H1C	DND1	CALHM1
ZNF609	SPIRE1	PPEF1	METTL21A	HIST1H1B	DNASE2B	CALD1
ZNF600	SPINK1	PPBP	METTL11B	HILPDA	DNASE1L2	CALCB
ZNF587	SPINDOC	PPAT	METRNL	HIGD2B	DNASE1	CALCA
ZNF563	SPIB	PPARGC1A	MET	HIGD2A	DNAJC9	CALB2
ZNF560	SPG21	PPARG	MEST	HIGD1A	DNAJC4	CADPS
ZNF552	SPERT	PPA1	MESP2	HIC2	DNAJC12	CADM1
ZNF541	SPDYE8P	POU6F2	MESP1	HIC1	DNAJC10	CACYBP
ZNF528	SPDYE6	POU5F1B	MEP1A	HIBCH	DNAJB13	CACNG7
ZNF521	SPDYC	POU4F3	MELTF	HHIPL2	DNAJB1	CACNG4
ZNF492	SPDYA	POU4F1	MELK	HHIP	DNAH2	CACNA1D
ZNF488	SPDEF	POU3F2	MEIOC	HHEX	DNAH17	CACNA1B
ZNF48	SPCS3	POU2F2	MEGF9	HGH1	DNAH14	CABYR
ZNF469	SPC25	POU2AF1	MEGF8	HESX1	DNAH11	CABP7
ZNF468	SPC24	POP5	MEFV	HES7	DNAAF3	CABP4
ZNF467	SPATA9	POP4	MEF2C	HES6	DNA2	CABLES2
ZNF443	SPATA25	POMP	MED7	HES4	DMTN	CA9
ZNF442	SPATA12	POLR3K	MED13	HES1	DMRTC2	CA8
ZNF439	SPAG4	POLR2J2	MED12L	HERC6	DMRTA2	CA4
ZNF432	SP7	POLR2I	MED10	HERC5	DMRT2	CA12
ZNF395	SP6	POLR2H	MED1	HEPHL1	DMRT1	C9orf50
ZNF324	SP140	POLQ	ME3	HEPACAM2	DMC1	C9orf116
ZNF322	SOX9	POLN	ME1	HENMT1	DMBX1	C8orf76
ZNF320	SOX7	POLE2	MDM2	HELLS	DLX6	C7orf61
ZNF30	SOX5	POLD1	MDK	HECW2	DLX5	C6orf52
ZNF292	SOX30	POLA2	MDH1	HEATR6	DLX4	C6orf223
ZNF286A	SOX2	POGLUT2	MDGA2	HEATR4	DLX2	C6orf15
ZNF280A	SOX18	POGK	MCOLN3	HDC	DLL4	C5orf34
ZNF239	SOX17	POFUT1	MCM8	HCN4	DLK1	C5AR2
ZNF222	SOX12	PODXL2	MCM7	HCN3	DLGAP5	C5AR1
ZNF22	SOX11	PODXL	MCM6	HCN2	DLGAP3	C3orf18
ZNF217	SOX10	PODN	MCM5	HCLS1	DLEU7	C3AR1
ZNF213	SOSTDC1	POC1B	MCM4	HCK	DLC1	C3
ZNF205	SORL1	POC1A	MCM3AP	HCFC1R1	DISP3	C2CD6
ZNF165	SORCS3	PNPLA8	MCM3	HCAR1	DISC1	C2CD4D
ZNF135	SORBS2	PNPLA7	MCM2	HBEGF	DIRAS2	C21orf58
ZNF117	SOD1	PNPLA6	MCM10	HAVCR2	DIRAS1	C20orf204
ZMIZ2	SOCS1	PNOC	MCF2L2	HAVCR1	DIPK2B	C1S
ZIC5	SNX9	PNMA3	MCCC2	HAUS8	DIPK1B	C1R
ZIC4	SNX31	PNMA2	MC1R	HAUS6	DIAPH3	C1QTNF6
ZIC3	SNX24	PNLDC1	MBP	HAUS3	DHX58	C1QTNF3
ZIC2	SNX22	PNKP	MBOAT7	HASPIN	DHX34	C1QTNF12
ZIC1	SNX13	PNCK	MBLAC1	HAPLN3	DHRS7	C1QL4
ZGRF1	SNURF	PMPCB	MBL2	HAL	DHRS11	C1QL1
ZGLP1	SNTG1	PMFBP1	MB	HAGHL	DHFR	C1QC
ZFYVE1	SNRPN	PMEPA1	MATN2	HADHA	DGLUCY	C1QB
ZFR2	SNRPE	PMEL	MASTL	H3F3A	DGKZ	C1QA
ZFP69B	SNRPD1	PMCH	MAST1	H2BFM	DGKI	C1orf61
ZFP36L2	SNRPC	PM20D1	MASP1	H2AFY2	DGKH	C1orf54
ZFP36L1	SNRPA1	PLXNC1	MARCO	H2AFY	DGKA	C1orf162

ZEB2	SNRNP40	PLXNA3	MARCKSL1	H2AFX	DGCR6	C1orf159
ZEB1	SNRNP25	PLXNA2	MARCH3	H2AFB1	DGAT2	C1orf112
ZDHHC9	SNORC	PLXNA1	MAPRE3	GZMM	DEPP1	C1GALT1C1
ZDHHC23	SNN	PLVAP	MAPK8IP2	GZMK	DEPDC5	C19orf57
ZCRB1	SNF8	PLSCR1	MAPK8IP1	GZMH	DEPDC1B	C19orf53
ZCCHC12	SNCG	PLPP5	MAPK7	GZMB	DEPDC1	C19orf48
ZC3HC1	SNCA	PLPP2	MAPK15	GZMA	DENND5B	C19orf25
ZC3H7A	SNAP47	PLP1	MAP9	GYPE	DENND1C	C17orf99
ZC3H3	SMYD2	PLOD3	MAP7D2	GUSB	DENND1B	C17orf64
ZBTB8B	SMS	PLLP	MAP4K2	GUCY2D	DEGS2	C17orf53
ZBTB41	SMPDL3B	PLK4	MAP4K1	GUCA1A	DEFA4	C12orf75
ZBTB32	SMPD3	PLK1	MAP3K13	GTSF1	DEDD	C12orf45
ZBTB18	SMN1	PLIN5	MAP3K1	GTSE1	DDX60	C11orf80
ZBTB10	SMIM10L2B	PLIN2	MAOB	GTF3C5	DDX58	C11orf53
ZBP1	SMIM10L2A	PLEKHO2	MANEAL	GTF3A	DDX53	C11orf45
ZAP70	SMG5	PLEKHH2	MANEA	GTF2F2	DDX49	C10orf82
YME1L1	SMG1	PLEKHG4	MAN1B1	GSTCD	DDX41	C10orf62
YJEFN3	SMCO3	PLEKHG3	MAN1A1	GSTA4	DDX4	BZW2
YIPF2	SMC5	PLEKHF2	MALL	GSS	DDX3Y	BUB3
YIF1B	SMC4	PLEKHF1	MAL	GSG1	DDX3X	BUB1B
YEATS4	SMC2	PLEKHB2	MAK	GRPR	DDX39A	BUB1
YEATS2	SMC1B	PLEKHB1	MAJIN	GRM8	DDX17	BTNL8
YBX2	SMAD4	PLEKHA4	MAGOHB	GRM5	DDX11	BTN3A2
YBX1	SMAD2	PLEK2	MAGED4	GRM2	DDR1	BTN2A2
YAE1	SMAD1	PLEK	MAGED2	GRM1	DDIT3	BTLA
XXYLT1	SLITRK6	PLD5	MAGED1	GRK7	DDIAS	BTG3
XRCC6	SLITRK1	PLD4	MAGEC3	GRINA	DCUN1D5	BST2
XRCC3	SLIT1	PLCL2	MAGEC2	GRIN3A	DCSTAMP	BST1
XRCC2	SLFN13	PLCH2	MAGEC1	GRIN2D	DCST2	BSND
XPR1	SLF1	PLCG2	MAGEA6	GRIN2B	DCST1	BSN
XPOT	SLCO5A1	PLCB4	MAGEA3	GRID2	DCN	BRSK2
XPO6	SLCO4C1	PLBD1	MAGEA2	GREM2	DCLK3	BRMS1
XKRX	SLCO2B1	PLAUR	MAGEA12	GREM1	DCLK2	BRIX1
XKR7	SLCO2A1	PLAU	MAGEA11	GRB7	DCAF8L2	BRIP1
XKR4	SLCO1C1	PLAGL2	MAGEA10	GRB2	DCAF4L2	BRINP2
XCL2	SLC9C1	PLAG1	MAFB	GRB10	DCAF4L1	BRINP1
XCL1	SLC9A5	PLAC9	MAFA	GRAP2	DCAF15	BRF2
XBP1	SLC7A8	PLAC8L1	MAD2L2	GPX2	DCAF13	BRDT
WWP1	SLC7A7	PLA2G7	MAD2L1	GPT2	DCAF10	BRCA2
WRAP73	SLC7A6	PLA2G4F	MACC1	GPSM1	DBX1	BRCA1
WNT7B	SLC7A5	PLA2G4C	MAB21L3	GPRIN1	DBNDD1	BRAP
WNT7A	SLC7A11	PLA2G4A	M1AP	GPRC5B	DBF4	BRAF
WNT5B	SLC7A10	PLA2G2D	LYZ	GPR89B	DAZAP2	BPIFA2
WNT10B	SLC6A4	PLA1A	LYVE1	GPR65	DAXX	BPI
WIPI2	SLC6A3	PKP4	LYST	GPR61	DARS	BPGM
WIPF1	SLC52A2	PKMYT1	LYPD6B	GPR45	DAPP1	BORA
WFIKKN1	SLC4A8	PKM	LYG2	GPR4	DAPK2	BOP1
WFDC5	SLC45A3	PKD2L2	LYG1	GPR27	DAPK1	BOLA2
WFDC10A	SLC45A2	PJVK	LY96	GPR26	DAP3	BMX
WDR97	SLC44A5	PJA2	LY9	GPR25	DAGLB	BMPR1B
WDR91	SLC44A2	PIWIL4	LY86	GPR19	DACH1	BMP6
WDR87	SLC39A6	PIWIL3	LY6G5C	GPR183	DAB2IP	BMP2K
WDR86	SLC39A14	PIWIL2	LUZP2	GPR18	DAB2	BMI1
WDR83	SLC38A8	PISD	LUM	GPR179	DAB1	BLVRB

WDR76	SLC38A1	PIR	LUC7L3	GPR174	CYTH1	BLOC1S3
WDR66	SLC35G6	PIP5KL1	LTO1	GPR171	CYSLTR2	BLNK
WDR53	SLC35E3	PIP5K1A	LTK	GPR153	CYREN	BLM
WDR48	SLC35D3	PIP4P1	LTC4S	GPR152	CYP4Z1	BLK
WDR34	SLC35B3	PIMREG	LTBP2	GPR15	CYP4F3	BIVM
WDHD1	SLC31A2	PIM3	LTB	GPR146	CYP4F2	BIRC7
WASHC5	SLC30A8	PIM2	LTA	GPR143	CYP2W1	BIRC5
WASF1	SLC30A5	PILRB	LST1	GPR139	CYP2S1	BIRC3
WARS	SLC30A3	PILRA	LSP1	GPR137C	CYP27C1	BIRC2
VWF	SLC2A6	PIK3R3	LSM7	GPR1	CYP27B1	BIN2
VWA5B2	SLC2A1	PIK3R2	LSM4	GPNMB	CYP27A1	BIK
VWA5B1	SLC29A4	PIK3IP1	LSM2	GPM6B	CYP26C1	BHLHE41
VWA5A	SLC29A3	PIK3CD	LSM1	GPI	CYP1B1	BHLHE40
VTI1B	SLC29A2	PIGX	LSG1	GPC4	CYP11A1	BFSP1
VSTM2L	SLC29A1	PIGU	LRRTM1	GPC3	CYLD	BEX5
VSIR	SLC27A6	PIGO	LRRN4CL	GPC2	CYBRD1	BEST1
VSIG4	SLC26A8	PIGL	LRRN3	GPC1	CYBB	BEND5
VPS28	SLC26A6	PIF1	LRRN1	GPBP1	CYB5B	BEND4
VPREB3	SLC26A5	PIEZO1	LRRIQ4	GPATCH2	CXorf40A	BCL7A
VOPP1	SLC26A2	PICALM	LRRC56	GPAT4	CXCR6	BCL6B
VNN3	SLC25A6	PIAS3	LRRC52	GPAA1	CXCR5	BCL6
VNN2	SLC25A52	PI3	LRRC45	GOLT1B	CXCR3	BCL2L12
VNN1	SLC25A40	PHYHIPL	LRRC42	GOLM1	CXCR2	BCL2L10
VN1R1	SLC25A37	PHRF1	LRRC4	GOLGA8B	CXCR1	BCL2L1
VMAC	SLC25A35	PHOSPHO1	LRRC26	GOLGA8A	CXCL9	BCL2A1
VLDLR	SLC25A21	PHLDA1	LRRC25	GOLGA7B	CXCL5	BCL2
VIRMA	SLC24A5	PHKA1	LRRC14B	GOLGA6L1	CXCL3	BCL11B
VIM	SLC24A3	PHF7	LRP8	GNLY	CXCL16	BCL11A
VILL	SLC24A2	PHF24	LRP5L	GNG7	CXCL14	BCAT2
VHLL	SLC22A4	PHEX	LRP4	GNG11	CXCL13	BCAT1
VGLL3	SLC22A15	PHC3	LRP1	GNAT1	CXCL12	BCAP31
VGLL1	SLC1A7	PGPEP1L	LRMP	GNAS	CXCL11	BBS4
VEPH1	SLC1A3	PGP	LRIT3	GNAI1	CXCL10	BBS12
VEGFB	SLC18A2	PGLYRP1	LRIG1	GMPS	CXCL1	BBC3
VEGFA	SLC17A9	PGK2	LRGUK	GMNN	CWF19L1	BASP1
VCX3A	SLC17A5	PGGHG	LRG1	GMDS	CUZD1	BARX2
VCX	SLC16A8	PGF	LRFN5	GM2A	CTU1	BARX1
VCAN	SLC16A7	PGC	LRFN4	GLYATL2	CTTNBP2	BANK1
VCAM1	SLC16A6	PGAM4	LRFN1	GLUL	CTTN	BANF1
VASN	SLC16A3	PFN2	LRCH4	GLUD1	CTSZ	BAMBI
VASH2	SLC16A14	PFKP	LPIN3	GLT8D2	CTSW	BACH2
VASH1	SLC16A11	PFKM	LPGAT1	GLS2	CTSV	BACE2
VAPA	SLC15A4	PFKFB4	LPAR5	GLRB	CTSK	BAALC
VANGL2	SLC15A3	PFDN2	LPAR2	GLP1R	CTSG	B4GALT7
VAMP2	SLC15A2	PF4	LPAR1	GLOD4	CTSE	B4GALT6
VAC14	SLC12A8	PEX5L	LOXL4	GLIPR1	CTRL	B4GALT3
UTS2B	SLC12A6	PEX10	LOX	GLDN	CTPS2	B4GALNT1
UTP20	SLC12A5	PELO	LONRF1	GLDC	CTNS	B3GNT4
USP9Y	SLC12A2	PEG10	LNP1	GLB1L3	CTNND2	B3GAT1
USP40	SLC12A1	PECAM1	LMNB2	GK	CTNNA2	B3GALNT1
USP35	SLC11A1	PEAR1	LMNB1	GJD2	CTLA4	B2M
USP32	SLBP	PDZD11	LMF2	GJB7	CTCFL	AZU1
USP26	SLAMF9	PDXK	LMAN2L	GJB4	CTBS	AXL
USP18	SLAMF8	PDX1	LMAN2	GJB1	CTAG2	AXIN2

USO1	SLAMF1	PDRG1	LKAAEAR1	GJA3	CTAG1B	AVPR1B
USF1	SLA	PDPN	LITAF	GIPR	CSTL1	AURKB
USB1	SKP2	PDP1	LIPA	GINS4	CSTA	AURKA
UQCRFS1	SKP1	PDLIM4	LINGO3	GINS2	CST7	AUNIP
UQCRC1	SKAP2	PDK3	LINGO1	GINS1	CST1	ATP9A
UQCRB	SKAP1	PDK1	LIN9	GIMAP5	CSRP2	ATP8B4
UQCC3	SKA3	PDIA4	LIN7A	GIMAP1-GIMAP5	CSRP1	ATP8A2
UPK3A	SKA1	PDGFRL	LIN28B	GGT5	CSPG4	ATP7A
UPK2	SIX4	PDGFRB	LIN28A	GGT1	CSNK2A1	ATP6V1H
UPK1B	SIX3	PDGFRA	LIMS3	GGH	CSMD3	ATP6V1F
UNC79	SIX1	PDGFA	LIMS1	GGCT	CSMD2	ATP6V1D
UNC5D	SIT1	PDF	LIMK1	GFRA3	CSF3R	ATP6V1C2
UNC5A	SIRPG	PDE7B	LIME1	GFRA1	CSF2RA	ATP6V1B2
UNC13A	SIRPB1	PDE6C	LIMA1	GFPT2	CSF2	ATP6V1B1
UMODL1	SIRPA	PDE4B	LILRB5	GFOD2	CSF1R	ATP6V1A
ULK1	SIK1	PDE2A	LILRB4	GFOD1	CSF1	ATP6V0D2
ULBP2	SIGLEC9	PDE1C	LILRB2	GFM1	CSE1L	ATP6V0B
ULBP1	SIGLEC8	PDCD5	LILRA6	GFI1	CSAG3	ATP6AP2
UHRF1	SIGLEC6	PDCD2L	LILRA5	GEMIN6	CSAG2	ATP6AP1
UGT2B17	SIGLEC5	PDCD1LG2	LILRA4	GEMIN2	CSAG1	ATP5MG
UGT1A8	SIGLEC15	PDCD1	LILRA2	GDPD5	CRYGN	ATP5ME
UFSP1	SIGLEC14	PCSK9	LILRA1	GDPD4	CRYBB1	ATP5F1B
UCN	SIGLEC10	PCSK5	LIG1	GDPD1	CRYBA4	ATP5F1A
UCKL1	SIGLEC1	PCSK2	LHX9	GDF9	CRYAB	ATP1B3
UCHL1	SIAH1	PCP2	LHFPL4	GDF6	CRY2	ATP1A3
UBL3	SHROOM4	PCOLCE2	LHCGR	GDF15	CRX	ATP1A1
UBE2T	SHOX2	PCOLCE	LGSN	GDF1	CRTC3	ATP13A1
UBE2S	SHOC1	PCNX4	LGMN	GDE1	CRTC2	ATP10D
UBE2QL1	SHLD1	PCNX2	LGALS9	GDAP1	CRTAM	ATM
UBE2Q1	SHISA7	PCNX1	LGALS1	GDA	CRPPA	ATL2
UBE2L6	SHE	PCNA	LFNG	GCSH	CRISPLD2	ATIC
UBE2F	SHCBP1	PCM1	LEPROTL1	GCNT1	CRISPLD1	ATG7
UBE2D2	SHC1	PCLAF	LEF1	GCK	CRISP3	ATG4D
UBE2C	SHARPIN	PCGF2	LDLRAD3	GBX2	CRIP3	ATF7IP
UBD	SHANK3	PCGF1	LDHA	GBP3	CRIP2	ATF4
UBASH3A	SH3TC2	PCDHGC4	LDB3	GBP2	CRHR2	ATAD5
UBA52	SH3RF1	PCDHGB1	LDB2	GBA	CRH	ATAD3B
UBA2	SH3KBP1	PCDHGA8	LCTL	GATM	CREM	ATAD2
UBA1	SH3D19	PCDHB8	LCT	GATD1	CREG2	ASRGL1
UAP1L1	SH2D1B	PCDHB5	LCP2	GATA3	CREBZF	ASPSCR1
TYROBP	SH2D1A	PCDHB3	LCORL	GATA2	CREB5	ASPRV1
TYR	SH2B1	PCDHB2	LCN12	GASK1B	CREB3L4	ASPM
TYMS	SGSM1	PCDHB16	LCN10	GAS2L3	CREB3L1	ASPHD1
TYMP	SGPL1	PCDHB11	LCK	GAPDH	CREB1	ASPH
TYK2	SGO2	PCDHA8	LCE1C	GALR3	CRACR2B	ASNS
TXNRD2	SGO1	PCDHA7	LCE1B	GALR1	CR2	ASIC1
TXNRD1	SGMS1	PCDHA5	LBR	GALNTL6	CPXM2	ASH2L
TXK	SGK3	PCDHA4	LBH	GALNT17	CPXM1	ASGR2
TUT7	SGCB	PCDHA2	LAT	GALNT16	CPVL	ASGR1
TUSC1	SFXN3	PCDHA11	LARS2	GALNT14	CPT1B	ASF1B
TUBB8	SFXN1	PCDHA1	LAPTM5	GALNT11	CPSF4L	ASCL4
TUBB4A	SFN	PCDH19	LAPTM4B	GALC	CPSF1	ASCL2
TUBB3	SF3B4	PCDH18	LAP3	GAL3ST4	CPNE9	ASCL1

TUBB2B	SF1	PBXIP1	LAMTOR2	GAL3ST1	CPNE7	ASB8
TUBB	SEZ6L2	PBX4	LAMP5	GAGE2A	CPNE5	ASB2
TUBA3E	SEZ6L	PBX3	LAMP3	GAGE12J	CPNE2	ASB16
TUBA3D	SETMAR	PBRM1	LAMP2	GADD45GIP1	CPNE1	ASAH2
TTYH3	SETDB1	PBK	LAMC1	GADD45A	CPM	ARX
TTLL4	SETD7	PAXX	LAMB4	GAD1	CPLX2	ART5
TTK	SET	PAX7	LAMB1	GABRR1	CPLX1	ARSH
TTC7B	SESN3	PAX6	LAMA5	GABRQ	CPEB2	ARSG
TTC39A	SERTAD2	PAX5	LAIR2	GABRG3	CPE	ARRB1
TTC38	SERPINI1	PAX3	LAIR1	GABRD	CPD	ARNTL
TSTA3	SERPINF1	PASK	LAGE3	GABRB2	CPA3	ARNT
TSSK6	SERPINE2	PARVB	LAG3	GABRA3	COX7C	ARMT1
TSSK1B	SERPINA3	PARVA	LACTB2	GABPB1	COX7B2	ARMCX3
TSPAN7	SERPINA1	PARPBP	L1CAM	GABARAP	COX7B	ARMC12
TSPAN5	SERINC4	PARK7	KYNU	GAB3	COX6B1	ARL9
TSPAN15	SERINC3	PAQR6	KSR1	G6PD	COX6A1	ARL6
TSLP	SERINC1	PAQR5	KRTCAP2	G0S2	COTL1	ARL17A
TSKS	SERHL2	PAQR4	KRTAP1-5	FZR1	CORT	ARL1
TSHR	SERGEF	PAPSS2	KRT80	FZD8	CORO7	ARID5A
TSGA13	SEPTIN8	PANX2	KRT74	FZD6	CORO1A	ARID4B
TSC22D3	SEPTIN5	PAMR1	KRT23	FZD4	COQ9	ARID3A
TSC22D1	SEMA6B	PAM	KREMEN2	FZD3	COPZ2	ARHGEF39
TSACC	SEMA4A	PALMD	KRBA1	FZD2	COPE	ARHGEF38
TRPV6	SEMA3F	PALLD	KPNA7	FYN	COMMD5	ARHGEF15
TRPV2	SEMA3B	PAK5	KPNA2	FYB1	COMMD4	ARHGEF1
TRPS1	SEMA3A	PAFAH1B3	KNTC1	FXYD6	COLQ	ARHGAP8
TRPM8	SEM1	PADI4	KNOP1	FXYD3	COLEC12	ARHGAP29
TRPM6	SELPLG	PADI3	KNL1	FUT9	COLCA2	ARHGAP26
TRPM4	SELP	PACSIN1	KMT2A	FUT8	COL9A2	ARHGAP25
TRPM2	SELL	PACRG	KLRK1	FUT5	COL9A1	ARHGAP22
TROAP	SELE	PABPC1L2B	KLRG1	FUT4	COL8A2	ARHGAP19
TRMT12	SEC61G	PABPC1L2A	KLRF1	FUT1	COL6A3	ARHGAP15
TRMO	SEC31B	PABPC1L	KLRD1	FURIN	COL6A2	ARHGAP11A
TRIT1	SEC14L1	PABPC1	KLRC4-KLRK1	FUCA1	COL6A1	ARHGAP10
TRIP13	SDK1	P4HB	KLRC4	FTSJ3	COL5A3	ARFGEF3
TRIP12	SDCBP	P4HA1	KLRC3	FTL	COL5A2	AQP9
TRIM9	SCX	P2RY2	KLRC1	FSTL5	COL4A4	AQP3
TRIM74	SCUBE2	P2RY14	KLRB1	FSTL1	COL4A1	AQP12A
TRIM67	SCRT1	P2RY13	KLK5	FSIP1	COL3A1	APP
TRIM59	SCNN1D	P2RY10	KLK2	FSD1	COL2A1	APOO
TRIM37	SCNN1A	P2RX5	KLK15	FSCN1	COL26A1	APOLD1
TRIM36	SCNM1	P2RX1	KLK1	FRY	COL23A1	APOL6
TRIM24	SCN9A	OXGR1	KLHL7	FRMPD4	COL1A2	APOL3
TRIM17	SCN3A	OXCT1	KLHL4	FRMPD2	COL1A1	APOE
TRIM16L	SCIN	OTX2	KLHL35	FRMD8	COL14A1	APOD
TRIM11	SCHIP1	OTUD7A	KLHL3	FRMD4A	COL12A1	APOC1
TRIB2	SCGN	OTP	KLHL26	FRMD3	COL11A2	APOBEC3G
TRHDE	SCGB1D2	OSM	KLHL21	FRK	COCH	APOBEC3A
TREML4	SCG5	OSER1	KLHL17	FRAT2	COA6	APLP2
TREML2	SCG3	OSBPL3	KLHL14	FPR3	CNTNAP5	APLP1
TREML1	SCG2	OSBPL1A	KLHDC4	FPR2	CNTNAP4	APLN
TREM2	SCARF2	OSBPL10	KLHDC2	FPR1	CNTNAP2	APCDD1
TREM1	SCARB2	OSBP2	KLF9	FPGS	CNTN3	APBB2

TRAT1	SCARA3	ORM1	KLF5	FOXRED2	CNR2	APBA2
TRAPPC5	SCAMP5	ORC6	KLF12	FOXP3	CNR1	AP3S1
TRAPPC1	SCAMP3	ORC1	KLF1	FOXP1	CNPY4	AP3M2
TRANK1	SCAMP1	OR6B2	KIT	FOXN4	CNPY3	AP2S1
TRAIP	SBK1	OR5B12	KISS1R	FOXM1	CNN1	AP2M1
TRAF5	SBDS	OR52I1	KISS1	FOXJ1	CNIH2	AP1S1
TRAF4	SAV1	OR51E1	KIRREL3	FOXI3	CNGB3	AP1M1
TRAF3IP3	SASS6	OR2B6	KIRREL1	FOXI2	CNGB1	AOX1
TRAF3IP2	SASH3	OR1L8	KIR3DL2	FOXI1	CMTM2	AOC1
TRAF2	SARS2	OR1J4	KIR3DL1	FOXH1	CMTM1	ANXA5
TRAF1	SARDH	OR1J2	KIR2DL4	FOXG1	CMKLR1	ANXA4
TRADD	SAPCD2	OPRD1	KIR2DL3	FOXE3	CMA1	ANTXR1
TPX2	SAMSN1	OPCML	KIR2DL1	FOXD4L6	CLUAP1	ANP32E
TPTE	SAMD10	OPA1	KIFC2	FOXD4L5	CLTC	ANOS1
TPST1	SAMD1	ONECUT2	KIFC1	FOXD4L4	CLTB	ANO9
TPSB2	SAGE1	OLR1	KIF4B	FOXD4L1	CLSPN	ANO7
TPSAB1	SACM1L	OLIG1	KIF4A	FOXD4	CLPTM1L	ANO5
TPM2	SAC3D1	OLFML3	KIF3C	FOXD1	CLPSL2	ANLN
TPM1	S1PR5	OLFML2B	KIF2C	FOXA1	CLPSL1	ANKS3
TPK1	S1PR1	OIP5	KIF26B	FOSL1	CLNS1A	ANKRD9
TPI1	S100B	OGT	KIF24	FOSB	CLNK	ANKRD55
TPBG	S100A9	OGDHL	KIF23	FOLH1	CLN8	ANKRD39
TP63	S100A5	OGA	KIF21A	FNIP2	CLN6	ANKRD34B
TP53TG3B	S100A4	OFD1	KIF20A	FN1	CLK4	ANKRD13D
TP53RK	S100A12	ODC1	KIF1A	FLVCR2	CLIC6	ANKRD10
TP53INP1	S100A1	OCSTAMP	KIF18B	FLT4	CLIC2	ANKLE1
TP53I13	RYR1	OCIAD2	KIF18A	FLT3LG	CLECL1	ANKFN1
TOX4	RXRG	OAS3	KIF15	FLI1	CLEC9A	ANK1
TOX	RUVBL2	OAS2	KIF14	FLAD1	CLEC7A	ANGPTL2
TOPBP1	RUNX3	OAS1	KIF13B	FKBP6	CLEC5A	ANGPT4
TOP2A	RUNX2	NXPH4	KIF13A	FKBP4	CLEC4C	ANGPT2
TOP1MT	RUBCNL	NXF5	KIF11	FKBP1B	CLEC4A	ANGEL1
TONSL	RTTN	NXF2	KIAA1549L	FITM2	CLEC2L	ANAPC13
TOMM40L	RTP2	NVL	KIAA1328	FIGN	CLEC2D	ANAPC11
TNK2	RTL9	NUTM1	KIAA1324L	FIBIN	CLEC2B	AMZ1
TNIP3	RTL1	NUSAP1	KIAA1324	FHL2	CLEC1A	AMT
TNFSF9	RTKN2	NUP85	KIAA1257	FGR	CLEC17A	AMPD2
TNFSF8	RTEL1	NUP37	KIAA0319	FGL2	CLEC16A	AMPD1
TNFSF4	RTBDN	NUP210L	KHDC4	FGGY	CLEC14A	AMOTL2
TNFSF18	RSRC1	NUP210	KHDC1L	FGFR4	CLEC10A	AMIGO3
TNFSF15	RSPH14	NUP155	KEL	FGFBP2	CLDN9	AMH
TNFSF14	RSAD2	NUP107	KDR	FGF9	CLDN5	AMDHD2
TNFSF13	RRP9	NUF2	KDM6B	FGF7	CLDN25	AMACR
TNFSF10	RRP12	NUDT9	KDM4C	FGF3	CLDN20	ALPL
TNFRSF9	RRM2	NUDT8	KDM1A	FGF18	CLDN11	ALPK3
TNFRSF8	RRM1	NUDT16	KCTD6	FGF17	CLCNKB	ALOXE3
TNFRSF6B	RRAGD	NUDT1	KCTD19	FGF12	CLCN7	ALOX5AP
TNFRSF4	RPUSD1	NUDCD1	KCP	FGD5	CLCN5	ALOX5
TNFRSF25	RPS9	NUCB2	KCNV1	FGD4	CLC	ALOX15
TNFRSF1B	RPS7	NTS	KCNU1	FFAR2	CKS2	ALKBH6
TNFRSF1A	RPS6KL1	NTRK1	KCNT2	FEZ1	CKS1B	ALKBH3
TNFRSF18	RPS6KC1	NTN3	KCNQ3	FEV	CKAP5	ALG8
TNFRSF17	RPS26	NTN1	KCNN2	FES	CKAP4	ALG1L
TNFRSF13B	RPS24	NT5M	KCNN1	FERMT1	CKAP2L	ALG11

TNFRSF11B	RPS21	NT5DC3	KCNMB4	FEN1	CKAP2	ALDOA
TNFRSF11A	RPS2	NT5DC2	KCNK9	FDX1	CITED4	ALDH9A1
TNFRSF10C	RPRML	NT5C1B	KCNK5	FDPS	CIT	ALDH3A2
TNFAIP6	RPP38	NSUN7	KCNJ3	FDFT1	CIPC	ALDH1B1
TNFAIP3	RPP21	NSUN5	KCNJ15	FCSK	CIP2A	ALDH1A2
TNFAIP2	RPL39L	NSMF	KCNJ11	FCRLB	CIAO3	ALCAM
TNFAIP1	RPL21	NSMCE2	KCNIP1	FCRLA	CHTF18	AKT3
TMSB15A	RPL10L	NSMCE1	KCNH8	FCRL6	CHST7	AKR1E2
TMPRSS9	RPIA	NSD3	KCNH2	FCRL5	CHST2	AKR1C3
TMPRSS3	ROPN1B	NRXN3	KCNH1	FCRL4	CHST15	AKR1C1
TMIE	ROMO1	NRTN	KCNG3	FCRL3	CHRNB4	AKR1B10
TMEM97	ROBO4	NRP2	KCNG2	FCRL2	CHRNB2	AKNA
TMEM9	RNPS1	NRIP3	KCNG1	FCRL1	CHRNA5	AKAP4
TMEM8B	RNFT2	NRG4	KCNF1	FCN1	CHRM5	AK5
TMEM81	RNF43	NRG2	KCNE3	FCMR	CHRM3	AIM2
TMEM74	RNF208	NRG1	KCNB2	FCGR3B	CHML	AIFM2
TMEM65	RNF207	NRBP2	KCNA3	FCGR3A	CHL1	AIF1
TMEM63C	RNF19A	NR5A1	KBTBD12	FCGR2B	CHIT1	AICDA
TMEM38A	RNF185	NR4A3	KBTBD11	FCGR2A	CHI3L2	AHSA1
TMEM33	RNF183	NR4A2	KAZALD1	FCGR1B	CHI3L1	AHR
TMEM31	RNF175	NR2F6	KATNAL2	FCGR1A	CHEK2	AHNAK
TMEM270	RNF144B	NR2E3	KAT6B	FCER2	CHEK1	AHI1
TMEM259	RNF141	NR2E1	KAT6A	FCER1G	CHD5	AHCYL2
TMEM255B	RNF128	NR2C2AP	KANK2	FCER1A	CHD1L	AHCYL1
TMEM255A	RNF125	NR0B1	KAAG1	FCAR	CHAF1B	AGPAT5
TMEM223	RNF113B	NQO1	JUP	FBXW9	CHAF1A	AGO2
TMEM222	RNASEH2A	NPTX2	JTB	FBXO5	CHAD	AGAP6
TMEM213	RNASE6	NPR1	JPH3	FBXO47	CH25H	AGAP5
TMEM211	RNASE3	NPPA	JMJD4	FBXO43	CGB5	AGAP4
TMEM205	RNASE2	NPL	JCHAIN	FBXO41	CFTR	AFF3
TMEM198	RNASE10	NPIPB3	JAG2	FBXO30	CFP	AFAP1L2
TMEM178A	RMND1	NPIPB15	JAG1	FBXO27	CFLAR	AFAP1L1
TMEM161B	RMI2	NPFFR1	IZUMO2	FBXO17	CFL1	AEN
TMEM160	RLN2	NPEPL1	ITPRIPL2	FBXL8	CFHR3	ADRM1
TMEM156	RLBP1	NPDC1	ITM2A	FBXL6	CETN3	ADRB2
TMEM150C	RIPPLY3	NPB	ITK	FBXL20	CERS6	ADORA1
TMEM145	RIPOR2	NPAS3	ITIH6	FBXL17	CERS4	ADIRF
TMEM140	RIMS4	NPAS1	ITIH2	FBXL16	CERS1	ADGRL4
TMEM132A	RIMS2	NOXO1	ITGB8	FBN3	CEP78	ADGRG3
TMEM129	RILPL2	NOXA1	ITGB7	FBN2	CEP72	ADGRG1
TMEM119	RIBC2	NOX4	ITGB4	FBN1	CEP68	ADGRF5
TMEM116	RHPN1	NOX3	ITGB2	FBLN5	CEP57	ADGRE5
TMEM101	RHOU	NOX1	ITGB1	FBLN1	CEP55	ADGRE3
TMEFF2	RHOT2	NOTUM	ITGAX	FBLL1	CEP152	ADGRE2
TMED2	RHOJ	NOS3	ITGAM	FATE1	CEP131	ADGRE1
TMC6	RHOB	NOS2	ITGAL	FASN	CENPW	ADGRB2
TMBIM6	RHOA	NOP58	ITGA9	FASLG	CENPO	ADGRB1
TM9SF4	RHEBL1	NOP10	ITGA4	FARP1	CENPN	ADD2
TM6SF1	RHBG	NOMO3	ITGA2B	FAP	CENPM	ADCYAP1
TM4SF4	RHBDD3	NOMO2	ITGA11	FANCI	CENPL	ADCY9
TM4SF19	RGS4	NOL7	ISM2	FANCG	CENPK	ADCY4
TM4SF18	RGS3	NOL4	ISLR	FANCE	CENPI	ADCY1
TLX3	RGS20	NOL3	ISL2	FANCD2	CENPH	ADCK5
TLR9	RGS2	NOL11	ISG20	FANCB	CENPF	ADCK1

TLR8	RGS17	NODAL	ISG15	FANCA	CENPE	ADAT3
TLR7	RGS16	NOD2	ISCU	FAM9C	CENPA	ADAT2
TLR2	RGS13	NNT	IRX6	FAM90A1	CEMP1	ADARB1
TLR10	RGS11	NMUR2	IRX5	FAM78B	CEMIP	ADAR
TLR1	RGS1	NMRAL1	IRX3	FAM72D	CELSR3	ADAP2
TLL2	RGPD5	NME8	IRGC	FAM72B	CELF5	ADAMTSL1
TLE5	RGMB	NME3	IRF9	FAM72A	CELF4	ADAMTS3
TLE3	RGL3	NMBR	IRF8	FAM71F2	CELF3	ADAMTS20
TLCD1	RGL2	NMB	IRF7	FAM71E2	CEL	ADAMTS2
TKTL1	RGCC	NLRP8	IRF4	FAM71E1	CEACAM8	ADAMTS18
TK1	RFXANK	NLRP3	IRF1	FAM71D	CEACAM6	ADAMTS17
TIPIN	RFX6	NLRP2	IRAK3	FAM50A	CEACAM3	ADAMDEC1
TIPARP	RFC5	NLRP12	IQGAP3	FAM49A	CEACAM19	ADAM8
TINAGL1	RFC4	NLRP11	IQCB1	FAM47C	CDT1	ADAM7
TIMM8A	RFC3	NLGN1	IPO11	FAM43A	CDKN3	ADAM33
TIMM50	REXO2	NKG7	IPCEF1	FAM3C	CDKN2AIP	ADAM28
TIMM13	RETREG1	NKD1	INTS8	FAM3B	CDKN2A	ADAM23
TIMELESS	RETNLB	NKAIN3	INTS7	FAM24B	CDKAL1	ADAM21
TIMD4	RET	NKAIN2	INTS4	FAM234B	CDK6	ADAM15
TIGIT	REPS2	NIPSNAP3B	INSYN2A	FAM222A	CDK5RAP3	ADAM12
TIGD5	REPS1	NIPSNAP3A	INSYN1	FAM189B	CDK5RAP2	ACVRL1
TIGD3	RENBP	NINJ1	INSR	FAM189A1	CDK5R1	ACVR1C
TIE1	REN	NID2	INSM1	FAM186B	CDK5	ACTR3B
TICRR	REM2	NIBAN3	INPPL1	FAM184B	CDK2	ACTR3
THY1	RELN	NHSL2	INPP5F	FAM178B	CDK18	ACTN4
THUMPD2	RELL2	NHLRC1	INPP4B	FAM174B	CDK16	ACTL8
THUMPD1	RELA	NHLH1	INO80C	FAM171A2	CDK12	ACTL6A
THSD7B	REG4	NFKBIA	INKA2	FAM170A	CDK10	ACTL10
THOC6	RECQL4	NFE4	ING3	FAM166A	CDK1	ACTG1
THEMIS2	REC8	NFE2L3	INA	FAM153B	CDIP1	ACTA2
THEMIS	RDX	NFE2L2	IMPG1	FAM136A	CDHR1	ACSS3
THEM6	RDM1	NFE2	IMPDH2	FAM135B	CDH8	ACSM1
THEG	RCSD1	NFATC4	IMPDH1	FAM133A	CDH7	ACSL3
THBS4	RCOR3	NFATC3	IMP3	FAM124B	CDH5	ACSL1
THBS2	RCOR2	NFATC2IP	ILF2	FAM117B	CDH4	ACRV1
THBS1	RCN3	NFATC1	IL9R	FAM111B	CDH24	ACP6
THBD	RCC2	NFAT5	IL9	FAM110B	CDH2	ACP5
THADA	RCC1	NF2	IL7R	FAM102A	CDH19	ACOT9
TGM5	RCAN3	NEUROD2	IL7	FADS1	CDH12	ACKR2
TGM3	RBPJL	NEUROD1	IL6ST	FADD	CDH1	ACHE
TGM2	RBPJ	NEURL2	IL5RA	FABP6	CDCA8	ACBD7
TGIF2LX	RBP3	NEU1	IL5	FABP5	CDCA7	ACAP1
TGIF1	RBP1	NETO2	IL4R	FABP4	CDCA5	ACAN
TGFBR2	RBMS3	NEMP2	IL4I1	FABP3	CDCA4	ACADM
TFRC	RBM5	NELFCD	IL4	FABP1	CDCA3	ACADL
TFPT	RBM3	NEK5	IL3RA	FAAP24	CDCA2	ACAD8
TFPI	RBM20	NEK3	IL37	F8A1	CDC7	ACACB
TFF2	RBIS	NEK2	IL34	F8	CDC6	ACACA
TFF1	RBCK1	NEIL3	IL32	F3	CDC5L	ABTB1
TFEC	RBBP8NL	NEFL	IL3	F2RL3	CDC45	ABT1
TFDP3	RAX	NEDD4L	IL2RB	F2RL2	CDC25C	ABI3BP
TFCP2L1	RASSF5	NECTIN3	IL2RA	F13A1	CDC25B	ABHD5
TFAP2E	RASSF4	NECTIN2	IL26	F12	CDC25A	ABHD3
TFAP2B	RASIP1	NDUFS8	IL22	F11R	CDC20B	ABHD2

TEX45	RASGRP3	NDUFS6	IL21R	EZR	CDC20	ABHD17A
TEX38	RASGRP2	NDUFB9	IL21	EZH2	CDC14A	ABHD16B
TEX264	RASGRP1	NDUFAF6	IL1RN	EYS	CDA	ABHD11
TEX19	RASGRF2	NDUFA8	IL1RL1	EYA2	CD96	ABCG2
TEX14	RASA4	NDUFA7	IL1RAPL1	EXT2	CD93	ABCD1
TESMIN	RASA3	NDUFA6	IL1B	EXOSC9	CD9	ABCC5
TESC	RASA1	NDUFA12	IL1A	EXOSC8	CD8B	ABCC4
TERT	RARRES2	NDRG1	IL18RAP	EXOSC7	CD8A	ABCC11
TEP1	RARA	NDFIP2	IL18R1	EXOSC6	CD86	ABCC1
TENM1	RAPGEF6	NDC80	IL18	EXOSC5	CD84	ABCB9
TEKT5	RAP1GAP	NCR3	IL17REL	EXOC6	CD80	ABCB5
TEKT2	RANGRF	NCR1	IL17A	EXOC2	CD79B	ABCB4
TEK	RANBP17	NCOA4	IL16	EXO5	CD79A	ABCA13
TEDC2	RANBP1	NCKAP1L	IL12RB2	EXO1	CD74	AARD
TEDC1	RAMP3	NCF2	IL12B	EWSR1	CD72	A4GNT
TECPR2	RAMP2	NCF1	IL12A	EVI5	CD70	
TEC	RALGPS2	NCBP2	IL10RB	EVI2B	CD7	
TEAD2	RALGAPA2	NCAPH	IKZF1	EVI2A	CD69	
TDRD6	RALB	NCAPG2	IKBKG	ETV4	CD68	
TDRD5	RAI14	NCAPG	IKBIP	ETV3	CD63	
TDRD1	RAE1	NCAPD2	IGSF8	ETV1	CD6	
TCTN3	RADX	NCAM2	IGSF6	ETS1	CD59	
TCTN2	RAD9A	NCALD	IGLON5	ESRRA	CD58	
TCP11	RAD54L	NBPF6	IGLL5	ESM1	CD55	
TCL1B	RAD54B	NBPF4	IGHMBP2	ESCO2	CD53	
TCL1A	RAD51AP1	NBPF15	IGFLR1	ESAM	CD52	
TCF7	RAD21	NAXE	IGFBP6	ERP27	CD5	
TCF19	RACGAP1	NAV3	IGFBP5	ERICH5	CD48	
TCF15	RAC3	NAV1	IGFBP3	ERI1	CD47	
TBXT	RABEP2	NATD1	IGF2BP3	ERG	CD40LG	
TBXAS1	RAB6B	NAT10	IGF2BP2	ERCC6L	CD40	
TBXA2R	RAB42	NAT1	IGF2BP1	ERC2	CD4	
TBX6	RAB40B	NARF	IGF2	ERBB3	CD3G	
TBX21	RAB3C	NANOS3	IGF1R	ERBB2	CD3E	
TBX2	RAB3B	NADSYN1	IGF1	EQTN	CD3D	
TBRG4	RAB3A	NACC1	IGDCC3	EPYC	CD38	
TBCD	RAB39B	NAB1	IFRD1	EPS8	CD37	
TBCC	RAB38	NAALADL2	IFNG	EPOR	CD36	
TBCB	RAB30	NAALADL1	IFNA10	EPOP	CD34	
TBC1D9	RAB27B	NAAA	IFITM2	EPN2	CD33	
TBC1D5	RAB20	NAA38	IFIT5	EPHX4	CD320	
TBC1D4	RAB1A	NAA20	IFIT3	EPHB6	CD302	
TBC1D3H	RAB15	NAA10	IFIT2	EPHB4	CD300LF	
TBC1D3C	RAB11FIP4	MZT2A	IFIT1	EPHB3	CD300LB	
TBC1D3B	RAB11FIP1	MZT1	IFIH1	EPHB2	CD300E	
TBC1D31	QTRT1	MZB1	IFI6	EPHA8	CD300A	
TBC1D3	QRSL1	MYT1	IFI44L	EPHA1	CD2BP2	
TBC1D26	QRFPR	MYRIP	IFI44	EPDR1	CD28	
TBC1D24	QPCTL	MYPOP	IFI35	EPB41	CD274	
TBC1D14	QPCT	MYO7A	IFI30	EOMES	CD27	
TBC1D1	QDPR	MYO3A	IFI16	ENY2	CD248	
TASOR2	PYHIN1	MYO1H	IDO1	ENTPD6	CD247	
TAS2R38	PYDC1	MYO1G	IDH3A	ENTPD1	CD244	
TAS1R1	PYCR3	MYO1F	ID3	ENPP3	CD226	

TARDBP	PYCR1	MYO15B	ID1	ENPP1	CD22
TAPBP	PXK	MYO10	ICOSLG	ENO2	CD209
TAP1	PVRIG	MYL9	ICOS	ENKD1	CD207
TANGO2	PUS1	MYH7B	ICAM3	ENGASE	CD200
TAL1	PUF60	MYH4	ICAM2	ENG	CD2
TAGLN3	PTX4	MYEOV	ICA1	ENC1	CD1E
TAGLN	PTRH2	MYCT1	IBSP	EN2	CD1D
TAGAP	PTPRT	MYCN	IARS	EN1	CD1C
TAFA3	PTPRJ	MYCL	HYLS1	EMP3	CD1B
TAFA2	PTPRG	MYCBP	HYDIN	EMP1	CD1A
TAF7L	PTPRCAP	MYC	HYAL3	EMID1	CD19
TAF4B	PTPRC	MYBPHL	HYAL2	EME2	CD180
TAF4	PTPRB	MYBPC3	HVCN1	EME1	CD163
TAF2	PTPN7	MYBL2	HUS1B	EMCN	CD160
TAF15	PTPN6	MYBL1	HUNK	ELP3	CD151
TACSTD2	PTPN20	MYB	HTR2C	ELP2	CD14
TACC3	PTPN11	MXRA8	HTR2B	ELOVL7	CD109
TAC4	PTH2R	MXD3	HSPG2	ELOC	CD101
TAC3	PTGS2	MXD1	HSPD1	ELOB	CCZ1B
SYTL5	PTGS1	MX2	HSPBP1	ELOA3	CCT6B
SYT8	PTGIS	MX1	HSPB7	ELOA2	CCT6A
SYT5	PTGIR	MUC20	HSPB6	ELN	CCT5
SYT2	PTGFRN	MTX1	HSPB1	ELMO2	CCT3
SYT17	PTGER4	MTUS1	HSPA6	ELMO1	CCT2
SYT15	PTGER2	MTSS2	HSPA4	ELK3	CCR9
SYT10	PTGDS	MTNR1B	HSPA1B	ELFN2	CCR7
SYNJ1	PTGDR2	MTMR7	HSPA1A	ELF1	CCR6
SYNGR3	PTGDR	MTHFD2	HSF4	ELAVL2	CCR5
SYNE4	PTDSS2	MTHFD1L	HSF2BP	ELANE	CCR3
SYN3	PTDSS1	MTFR2	HSD17B3	EIF4A1	CCR2
SYCP2L	PTCRA	MTERF3	HSD17B11	EIF3L	CCR10
SYCP2	PTCHD4	MTCP1	HSD11B2	EIF3H	CCR1
SYCE3	PTBP1	MTBP	HSD11B1	EIF2S2	CCNO
SYCE2	PTAFR	MSRB1	HS6ST3	EIF2AK2	CCNI2
SYCE1L	PSTPIP1	MSR1	HS3ST2	EIF1B	CCNF
SYCE1	PSRC1	MSMP	HRK	EIF1	CCNE2
SVOPL	PSPN	MSLNL	HRH4	EID1	CCNE1
SVEP1	PSPH	MSL1	HRH2	EGR3	CCND2
SV2C	PSPC1	MSI1	HRH1	EGR2	CCND1
SUV39H1	PSMG3	MSH5	HPSE	EGLN3	CCNB3
SUSD3	PSMG1	MSH4	HPS4	EGFR	CCNB2
SULT4A1	PSME3	MSC	HPGDS	EGFL7	CCNB1
SULT1C4	PSMD4	MSANTD3	HPGD	EGFL6	CCNA2
SULT1C2	PSMD3	MS4A7	HPDL	EFNB1	CCNA1
SULF1	PSMD2	MS4A6A	HPCA	EFNA5	CCN4
SUB1	PSMC3IP	MS4A4A	HOXD1	EFNA4	CCN2
STXBP6	PSMB8	MS4A3	HOXC13	EFNA1	CCL8
STX4	PSMB4	MS4A2	HOXC12	EFEMP2	CCL7
STX1A	PSMA7	MS4A1	HOXB9	EFEMP1	CCL5
STX18	PSMA6	MRPS30	HOXB5	EFCAB13	CCL4L2
STX16	PSKH1	MRPS21	HOXB13	EDRF1	CCL4
STX10	PSG2	MRPS17	HOXA9	EDN1	CCL3L1
STRA8	PSAT1	MRPL55	HOXA4	EDARADD	CCL26
STRA6	PSAP	MRPL52	HOXA3	EDAR	CCL23

STPG4	PRX	MRPL47	HOXA2	EDA2R	CCL22
STPG3	PRSS50	MRPL4	HOXA1	ECT2	CCL21
STPG2	PRSS41	MRPL24	HORMAD1	ECSCR	CCL20
STON2	PRSS23	MROH7	HOMER2	ECE2	CCL2
STMN1	PRSS21	MRGPRE	HNRNPM	ECE1	CCL19
STK32A	PRRX2	MRGBP	HNRNPLL	EBI3	CCL18
STK31	PRRX1	MRC2	HNRNPH1	EBF2	CCL17
STIM1	PRR7	MRC1	HNRNPD	EBF1	CCL14
STIL	PRR5L	MPZL1	HNMT	EAF2	CCL13
STEAP4	PRR5	MPV17L2	HNF1B	E2F7	CCL1
STEAP1	PRR19	MPV17	HMSD	E2F5	CCKBR
STC2	PRR16	MPP6	HMMR	E2F3	CCDC91
STAT5B	PRR11	MPP1	HMGB3	E2F1	CCDC88A
STAT5A	PRPF4	MPO	HMGB2	DYTN	CCDC80
STAT4	PROSER1	MPL	HMGA1	DYSF	CCDC77
STAT3	PROK2	MPG	HMG20B	DYNLT1	CCDC74A
STAT1	PRODH	MPC2	HLA-G	DYNC2LI1	CCDC57
STARD3	PROCA1	MON1B	HLA-DRB1	DVL3	CCDC34
STAP1	PRMT1	MOCS3	HLA-DRA	DUT	CCDC28B
STAG3	PRLR	MOCOS	HLA-DQB1	DUSP6	CCDC183
STAC	PRKY	MOB1B	HLA-DQA2	DUSP5	CCDC18
STAB1	PRKRIP1	MNDA	HLA-DQA1	DUSP23	CCDC177
ST8SIA5	PRKCQ	MND1	HLA-DPB1	DUSP2	CCDC167
ST8SIA4	PRKCH	MMS22L	HLA-DPA1	DUSP15	CCDC154
ST8SIA2	PRKCG	MMRN2	HLA-DOB	DUSP14	CCDC151
ST8SIA1	PRKCD	MMRN1	HLA-DMB	DUSP12	CCDC146
ST6GALNAC4	PRKAG2	MMP9	HLA-DMA	DUS4L	CCDC134
ST6GALNAC2	PRIM1	MMP25	HLA-C	DUS1L	CCDC117
ST3GAL6	PRG2	MMP24	HLA-B	DTYMK	CCDC102B
ST3GAL5	PRF1	MMP2	HLA-A	DTX4	CC2D2B
ST3GAL4	PREP	MMP16	HK3	DTX1	CBX8
ST3GAL1	PRELP	MMP15	HJURP	DTNB	CBX6
ST18	PRELID3A	MMP12	HIVEP2	DTL	CBX3
SSX5	PRDM4	MMEL1	HIST4H4	DTD1	CBX2
SSX1	PRDM13	MME	HIST3H2BB	DSN1	CBWD6
SSUH2	PRCP	MMD	HIST3H2A	DSCC1	CBWD3
SSU72	PRC1	MLPH	HIST2H3D	DSC1	CBLN1
SSPO	PRAP1	MLANA	HIST2H3C	DRICH1	CBLB
SS18L2	PRAMEF4	MKRN3	HIST2H2AC	DRC7	CBARP
SRXN1	PRAME	MKI67	HIST1H4J	DRAXIN	CAVIN2
SRSF7	PRAC2	MITF	HIST1H4I	DPYSL5	CAV1
SRSF6	PQBP1	MIS18A	HIST1H4H	DPYD	CATSPERG
SRSF4	PPP4R3C	MIPEP	HIST1H4E	DPY19L1	CAT
SRSF12	PPP4R3A	MINPP1	HIST1H4D	DPT	CASQ1
SRSF1	PPP1R3C	MIF4GD	HIST1H4C	DPP7	CASP8
SRRM3	PPP1R35	MIF	HIST1H4B	DPP4	CASP5

Table S2. IMvigor 210 cohort.

Survival time in years	Survival state	Best confirmed overall response	Binary response	IC Level	TC Level	Immune phenotype	TMB	NEO
4,6324	1	PD	SD/PD	IC1	TC0	excluded	NA	NA
16,2300	1	PD	SD/PD	IC0	TC0	excluded	NA	NA
3,1211	1	PD	SD/PD	IC0	TC0	NA	NA	NA
21,1581	1	SD	SD/PD	IC1	TC0	NA	NA	NA
0,8214	1	NE	NA	IC0	TC0	inflamed	NA	NA
1,2156	1	NE	NA	IC0	TC0	NA	NA	NA
15,6057	1	SD	SD/PD	IC1	TC0	desert	NA	NA
7,9179	1	SD	SD/PD	IC0	TC0	NA	NA	NA
10,1848	1	PD	SD/PD	IC2+	TC2+	inflamed	NA	NA
4,9938	1	PD	SD/PD	IC0	TC0	NA	NA	NA
21,1581	1	SD	SD/PD	IC0	TC0	excluded	NA	NA
3,2197	1	PD	SD/PD	IC0	TC0	NA	NA	NA
2,2341	1	PD	SD/PD	IC0	TC0	NA	NA	NA
6,7023	1	PD	SD/PD	IC0	TC0	NA	NA	NA
0,5585	1	PD	SD/PD	IC1	TC0	excluded	NA	NA
8,7064	1	PD	SD/PD	IC0	TC0	desert	NA	NA
11,6632	1	SD	SD/PD	IC1	TC0	excluded	NA	NA
13,2731	1	PD	SD/PD	IC1	TC0	excluded	NA	NA
3,9097	1	PD	SD/PD	IC1	TC0	NA	NA	NA
1,9384	1	NE	NA	IC0	TC0	excluded	NA	NA
7,2279	1	PD	SD/PD	IC2+	TC0	excluded	NA	NA
1,4456	1	PD	SD/PD	IC0	TC0	NA	NA	NA
6,7351	1	SD	SD/PD	IC0	TC0	NA	NA	NA
1,3142	1	NE	NA	IC1	TC0	excluded	NA	NA
5,8480	1	PD	SD/PD	IC2+	TC0	excluded	NA	NA
2,5298	1	PD	SD/PD	IC2+	TC0	excluded	NA	NA
1,9055	1	NE	NA	IC1	TC0	excluded	NA	NA
0,8214	1	NE	NA	IC1	TC0	excluded	NA	NA
2,2998	1	PD	SD/PD	IC0	TC0	NA	NA	NA
12,8460	1	SD	SD/PD	IC0	TC0	NA	NA	NA
3,2526	1	PD	SD/PD	IC0	TC0	NA	NA	NA
6,5051	1	PD	SD/PD	IC0	TC0	inflamed	NA	NA
0,3614	1	NE	NA	IC2+	TC0	inflamed	NA	NA
4,3696	1	PD	SD/PD	IC1	TC0	NA	NA	NA
3,7454	1	PD	SD/PD	IC0	TC0	NA	NA	NA
2,9240	1	NE	NA	IC2+	TC1	NA	NA	NA
1,8398	1	NE	NA	IC1	TC2+	NA	NA	NA
1,9384	1	PD	SD/PD	IC1	TC0	NA	22	NA
9,2320	1	SD	SD/PD	IC0	TC0	desert	22	NA
4,9610	1	NE	NA	IC2+	TC0	inflamed	21	NA
1,4784	1	PD	SD/PD	IC0	TC0	desert	15	NA
0,5257	1	NE	NA	IC1	TC0	NA	15	NA
8,0821	1	PD	SD/PD	IC0	TC0	desert	14	NA
17,7741	1	SD	SD/PD	IC2+	TC0	excluded	14	NA
2,4641	1	NE	NA	IC0	TC0	excluded	14	NA
1,8727	1	NE	NA	IC1	TC0	desert	13	NA
3,8439	1	PD	SD/PD	IC2+	TC0	NA	12	NA
10,9076	1	PD	SD/PD	IC0	TC0	NA	12	NA
5,6509	1	PD	SD/PD	IC1	TC0	desert	10	NA
15,5400	1	PR	CR/PR	IC1	TC0	desert	10	NA

15,4086	1	SD	SD/PD	IC2+	TC2+	inflamed	10	NA
4,1725	1	PD	SD/PD	IC1	TC0	desert	9	NA
6,6037	1	PD	SD/PD	IC2+	TC0	excluded	9	NA
7,0637	1	NE	NA	IC1	TC0	NA	9	NA
3,8768	1	PD	SD/PD	IC2+	TC0	desert	8	NA
2,0370	1	NE	NA	IC2+	TC1	inflamed	8	NA
1,9713	1	NE	NA	IC2+	TC2+	excluded	8	NA
6,4066	1	SD	SD/PD	IC1	TC0	desert	8	NA
11,3676	1	SD	SD/PD	IC1	TC0	excluded	7	NA
9,2649	1	CR	CR/PR	IC2+	TC0	excluded	6	NA
17,9055	1	PD	SD/PD	IC2+	TC0	excluded	6	NA
2,0698	1	PD	SD/PD	IC1	TC0	desert	6	NA
3,7125	1	PD	SD/PD	IC1	TC0	excluded	6	NA
0,8871	1	NE	NA	IC0	TC0	desert	5	NA
6,7023	1	PD	SD/PD	IC1	TC0	excluded	5	NA
3,1869	1	PD	SD/PD	IC1	TC1	inflamed	5	NA
9,0349	1	PD	SD/PD	IC0	TC0	desert	5	NA
4,3696	1	PD	SD/PD	IC0	TC0	desert	5	NA
3,8768	1	PD	SD/PD	IC2+	TC2+	excluded	4	NA
0,5914	1	NE	NA	IC2+	TC0	excluded	3	NA
4,1396	1	SD	SD/PD	IC1	TC0	excluded	2	NA
8,0821	1	PD	SD/PD	IC1	TC2+	excluded	1	NA
1,7084	1	NE	NA	IC0	TC0	excluded	36	6,80
2,4969	1	PD	SD/PD	IC2+	TC2+	inflamed	44	6,20
15,6386	1	SD	SD/PD	IC1	TC0	excluded	18	4,69
10,4148	1	PD	SD/PD	IC1	TC0	excluded	32	4,10
17,0842	1	PD	SD/PD	IC2+	TC0	inflamed	21	3,82
13,4045	1	SD	SD/PD	IC2+	TC2+	inflamed	16	3,76
1,0513	1	NE	NA	IC2+	TC2+	inflamed	26	3,69
19,2854	1	PD	SD/PD	IC1	TC2+	NA	NA	3,57
4,2710	1	PD	SD/PD	IC1	TC0	desert	21	3,53
1,7413	1	PD	SD/PD	IC0	TC0	desert	NA	3,33
7,3265	1	PD	SD/PD	IC2+	TC2+	inflamed	19	3,24
1,1499	1	NE	NA	IC1	TC0	inflamed	32	3,22
17,0185	1	PR	CR/PR	IC2+	TC0	inflamed	49	2,71
7,7207	1	PD	SD/PD	IC1	TC0	excluded	14	2,71
0,2300	1	NE	NA	IC2+	TC2+	excluded	NA	2,67
6,2423	1	PD	SD/PD	IC2+	TC0	inflamed	14	2,55
5,0595	1	PD	SD/PD	IC1	TC0	excluded	15	2,24
10,8419	1	PD	SD/PD	IC0	TC0	NA	17	2,18
2,8912	1	NE	NA	IC1	TC0	inflamed	5	2,12
16,4600	1	PD	SD/PD	IC2+	TC2+	excluded	18	2,04
0,6242	1	NE	NA	IC2+	TC2+	inflamed	6	2,02
2,6283	1	PD	SD/PD	IC0	TC0	NA	18	2,00
2,1027	1	PD	SD/PD	IC1	TC2+	excluded	NA	1,92
6,8994	1	PD	SD/PD	IC1	TC0	NA	19	1,80
5,8809	1	PD	SD/PD	IC0	TC0	excluded	17	1,73
12,8131	1	PR	CR/PR	IC0	TC0	desert	5	1,73
5,1253	1	SD	SD/PD	IC2+	TC0	excluded	NA	1,53
4,5339	1	SD	SD/PD	IC2+	TC0	excluded	2	1,47
6,7023	1	SD	SD/PD	IC1	TC2+	excluded	6	1,45
7,4579	1	PD	SD/PD	IC0	TC0	excluded	5	1,45
6,2423	1	SD	SD/PD	IC1	TC0	excluded	NA	1,43
12,4189	1	PD	SD/PD	IC1	TC0	inflamed	5	1,43

2,2012	1	PD	SD/PD	IC0	TC0	desert	NA	1,41
3,4497	1	PD	SD/PD	IC1	TC0	desert	13	1,41
3,4825	1	PD	SD/PD	IC1	TC2+	inflamed	5	1,39
3,1211	1	PD	SD/PD	IC2+	TC0	inflamed	7	1,37
1,6756	1	NE	NA	IC1	TC1	excluded	7	1,37
4,8953	1	SD	SD/PD	IC0	TC0	desert	9	1,33
5,4867	1	PD	SD/PD	IC1	TC0	desert	7	1,33
10,4805	1	PD	SD/PD	IC0	TC0	excluded	11	1,29
2,6283	1	PD	SD/PD	IC2+	TC1	excluded	8	1,27
2,1355	1	NE	NA	IC2+	TC0	excluded	5	1,27
2,8912	1	PD	SD/PD	IC1	TC0	desert	13	1,25
0,6242	1	NE	NA	IC1	TC1	inflamed	10	1,25
6,9651	1	SD	SD/PD	IC0	TC0	desert	11	1,22
5,3881	1	SD	SD/PD	IC1	TC0	excluded	8	1,18
9,4949	1	PD	SD/PD	IC1	TC2+	desert	5	1,18
16,2628	1	PD	SD/PD	IC0	TC1	NA	5	1,18
1,4456	1	NE	NA	IC0	TC0	excluded	5	1,14
6,0123	1	PD	SD/PD	IC2+	TC0	desert	17	1,12
4,3696	1	PD	SD/PD	IC2+	TC0	inflamed	14	1,10
7,3922	1	SD	SD/PD	IC2+	TC0	inflamed	10	1,08
2,1355	1	PD	SD/PD	IC0	TC0	NA	7	1,08
2,1355	1	PD	SD/PD	IC1	TC0	excluded	NA	1,04
0,8542	1	NE	NA	IC1	TC0	excluded	9	1,02
0,4271	1	NE	NA	IC1	TC0	excluded	6	1,02
15,3758	1	SD	SD/PD	IC1	TC0	excluded	6	1,02
0,4928	1	NE	NA	IC1	TC0	excluded	5	1,00
0,4928	1	NE	NA	IC1	TC0	excluded	4	1,00
0,7556	1	NE	NA	IC2+	TC0	inflamed	NA	0,98
3,9754	1	PD	SD/PD	IC0	TC0	desert	14	0,98
0,6242	1	NE	NA	IC2+	TC2+	inflamed	5	0,98
9,5606	1	PD	SD/PD	IC0	TC0	desert	5	0,96
1,8070	1	NE	NA	IC2+	TC2+	inflamed	3	0,96
5,9466	1	PD	SD/PD	IC2+	TC0	inflamed	3	0,96
2,6612	1	PD	SD/PD	IC0	TC0	NA	5	0,94
10,5791	1	SD	SD/PD	IC2+	TC0	inflamed	15	0,92
1,1170	1	PD	SD/PD	IC1	TC1	excluded	6	0,92
12,8460	1	PD	SD/PD	IC2+	TC0	inflamed	5	0,90
2,7598	1	PD	SD/PD	IC2+	TC2+	excluded	8	0,88
11,4004	1	PR	CR/PR	IC2+	TC2+	desert	NA	0,86
2,1684	1	PD	SD/PD	IC2+	TC2+	inflamed	17	0,86
5,6509	1	PD	SD/PD	IC1	TC0	inflamed	4	0,86
2,5298	1	PD	SD/PD	IC1	TC2+	desert	NA	0,84
6,2752	1	PD	SD/PD	IC2+	TC0	inflamed	14	0,84
10,5462	1	PD	SD/PD	IC2+	TC0	inflamed	3	0,84
6,7351	1	PD	SD/PD	IC2+	TC0	excluded	6	0,82
10,3491	1	PD	SD/PD	IC1	TC0	desert	7	0,80
2,7269	1	PD	SD/PD	IC1	TC0	excluded	10	0,78
1,6756	1	PD	SD/PD	IC0	TC0	excluded	11	0,76
0,3614	1	NE	NA	IC0	TC0	NA	12	0,75
14,7515	1	PD	SD/PD	IC0	TC0	desert	7	0,75
3,8768	1	PD	SD/PD	IC2+	TC1	inflamed	3	0,75
10,2505	1	SD	SD/PD	IC2+	TC2+	NA	NA	0,73
1,0185	1	PD	SD/PD	IC0	TC0	NA	12	0,73
2,0698	1	PD	SD/PD	IC0	TC0	desert	8	0,73

3,6140	1	PD	SD/PD	IC0	TC0	excluded	5	0,73
8,2793	1	SD	SD/PD	IC1	TC0	NA	NA	0,71
11,1047	1	PD	SD/PD	IC1	TC0	excluded	3	0,71
3,5483	1	PD	SD/PD	IC2+	TC0	NA	6	0,69
2,4312	1	PD	SD/PD	IC1	TC0	excluded	5	0,67
8,8378	1	PD	SD/PD	IC1	TC0	excluded	5	0,65
5,5195	1	PD	SD/PD	IC1	TC0	excluded	2	0,65
2,1355	1	PD	SD/PD	IC0	TC0	NA	NA	0,61
3,6140	1	PD	SD/PD	IC2+	TC2+	excluded	3	0,61
9,8563	1	PD	SD/PD	IC1	TC0	excluded	0	0,61
7,6222	1	PD	SD/PD	IC2+	TC2+	inflamed	12	0,59
1,5770	1	NE	NA	IC1	TC1	NA	3	0,57
20,7639	1	PD	SD/PD	IC0	TC0	excluded	7	0,55
3,5154	1	PD	SD/PD	IC1	TC0	desert	6	0,55
3,1211	1	PD	SD/PD	IC0	TC0	desert	7	0,53
5,3881	1	PD	SD/PD	IC2+	TC1	excluded	5	0,53
8,2464	1	SD	SD/PD	IC0	TC1	NA	5	0,53
2,6940	1	NE	NA	IC2+	TC0	excluded	4	0,53
15,8029	1	PD	SD/PD	IC0	TC0	excluded	10	0,51
4,5010	1	PD	SD/PD	IC0	TC0	desert	6	0,51
2,5955	1	PD	SD/PD	IC1	TC0	desert	NA	0,49
7,8522	1	PD	SD/PD	IC2+	TC2+	desert	3	0,49
0,8542	1	PD	SD/PD	IC1	TC0	excluded	6	0,47
9,0021	1	PD	SD/PD	IC2+	TC1	excluded	5	0,47
7,8850	1	PD	SD/PD	IC1	TC0	excluded	8	0,45
1,8070	1	PD	SD/PD	IC0	TC0	excluded	3	0,45
7,9507	1	SD	SD/PD	IC0	TC0	desert	NA	0,43
17,9713	1	PD	SD/PD	IC1	TC0	excluded	NA	0,43
11,2690	1	PD	SD/PD	IC1	TC0	desert	9	0,43
19,1211	1	SD	SD/PD	IC1	TC0	desert	6	0,43
2,5626	1	PD	SD/PD	IC0	TC0	excluded	6	0,43
2,6940	1	NE	NA	IC0	TC0	excluded	5	0,43
9,8891	1	PD	SD/PD	IC2+	TC2+	excluded	5	0,43
5,6838	1	PD	SD/PD	IC0	TC0	desert	11	0,41
1,4127	1	PD	SD/PD	IC0	TC0	desert	12	0,39
15,8686	1	PD	SD/PD	IC2+	TC0	inflamed	7	0,39
2,8255	1	NE	NA	IC0	TC0	desert	5	0,39
3,5483	1	SD	SD/PD	IC1	TC2+	excluded	7	0,33
5,7166	1	PD	SD/PD	IC1	TC0	excluded	5	0,33
21,0267	1	SD	SD/PD	IC1	TC0	desert	5	0,33
2,7598	1	PD	SD/PD	IC1	TC0	excluded	14	0,31
5,8809	1	PD	SD/PD	IC0	TC0	desert	13	0,31
8,7721	1	PD	SD/PD	IC1	TC0	desert	1	0,31
2,1027	1	PD	SD/PD	IC1	TC0	desert	13	0,29
8,0164	1	SD	SD/PD	IC1	TC1	excluded	5	0,29
8,0821	1	SD	SD/PD	IC2+	TC2+	excluded	4	0,29
13,2731	1	SD	SD/PD	IC0	TC0	desert	3	0,29
5,8480	1	PD	SD/PD	IC2+	TC0	excluded	8	0,27
11,9261	1	PD	SD/PD	IC2+	TC0	excluded	7	0,27
6,1109	1	PD	SD/PD	IC1	TC0	excluded	5	0,27
5,3881	1	PD	SD/PD	IC1	TC0	excluded	5	0,27
2,8255	1	NE	NA	IC0	TC0	NA	3	0,27
5,3881	1	SD	SD/PD	IC0	TC0	excluded	1	0,27
7,9507	1	PD	SD/PD	IC1	TC0	NA	4	0,25

2,2341	1	PD	SD/PD	IC2+	TC0	desert	5	0,24
1,7084	1	NE	NA	IC1	TC0	excluded	4	0,24
10,8747	1	PD	SD/PD	IC2+	TC2+	excluded	NA	0,22
13,3388	1	PD	SD/PD	IC1	TC0	excluded	11	0,22
14,1273	1	PD	SD/PD	IC1	TC0	inflamed	6	0,22
1,1828	1	PD	SD/PD	IC1	TC1	NA	2	0,22
1,8070	1	NE	NA	IC0	TC0	desert	1	0,22
10,4805	1	PD	SD/PD	IC1	TC0	inflamed	NA	0,20
2,2341	1	PD	SD/PD	IC0	TC0	excluded	NA	0,20
8,0164	1	PD	SD/PD	IC0	TC0	desert	3	0,20
1,9713	1	PD	SD/PD	IC2+	TC2+	inflamed	1	0,20
6,8008	1	PD	SD/PD	IC0	TC0	NA	NA	0,16
12,7146	1	SD	SD/PD	IC1	TC0	inflamed	4	0,16
15,3101	1	SD	SD/PD	IC0	TC0	desert	2	0,16
2,8583	1	PD	SD/PD	IC1	TC0	desert	4	0,14
2,1355	1	PD	SD/PD	IC1	TC0	desert	5	0,12
9,7577	1	PD	SD/PD	IC1	TC2+	excluded	5	0,10
3,1211	1	PD	SD/PD	IC1	TC0	excluded	2	0,06
13,3060	1	SD	SD/PD	IC2+	TC0	inflamed	NA	0,04
10,1191	1	SD	SD/PD	IC2+	TC2+	inflamed	1	0,04
1,7084	0	SD	SD/PD	IC0	TC0	NA	NA	NA
22,1109	0	PD	SD/PD	IC1	TC0	excluded	NA	NA
22,1109	0	PR	CR/PR	IC0	TC0	desert	NA	NA
22,1109	0	SD	SD/PD	IC0	TC0	excluded	NA	NA
18,1684	0	PD	SD/PD	NA	NA	NA	NA	NA
21,2567	0	CR	CR/PR	IC2+	TC0	excluded	NA	NA
20,6982	0	SD	SD/PD	IC1	TC0	NA	NA	NA
20,7310	0	CR	CR/PR	IC2+	TC0	excluded	NA	NA
18,6612	0	SD	SD/PD	IC1	TC0	excluded	NA	NA
16,4600	0	PD	SD/PD	IC0	TC0	desert	NA	NA
16,0329	0	SD	SD/PD	IC0	TC0	NA	NA	NA
13,9959	0	PD	SD/PD	IC0	TC0	NA	NA	NA
0,6899	0	NE	NA	IC1	TC0	NA	50	NA
20,0739	0	CR	CR/PR	IC2+	TC0	excluded	35	NA
18,1684	0	PR	CR/PR	IC1	TC0	excluded	32	NA
20,2710	0	CR	CR/PR	IC2+	TC0	inflamed	24	NA
22,7351	0	PR	CR/PR	IC2+	TC0	excluded	19	NA
22,4066	0	PR	CR/PR	IC0	TC1	desert	18	NA
20,6324	0	PR	CR/PR	IC1	TC0	inflamed	14	NA
0,6242	0	NE	NA	IC1	TC0	desert	14	NA
0,8214	0	NE	NA	IC0	TC0	desert	13	NA
21,6509	0	PR	CR/PR	IC0	TC0	NA	12	NA
24,4764	0	PR	CR/PR	IC1	TC0	excluded	9	NA
20,0739	0	CR	CR/PR	IC2+	TC0	inflamed	8	NA
3,4497	0	PD	SD/PD	IC0	TC0	excluded	5	NA
20,7310	0	SD	SD/PD	IC1	TC0	desert	5	NA
2,1027	0	PD	SD/PD	IC1	TC0	excluded	5	NA
2,1027	0	PD	SD/PD	IC2+	TC0	excluded	4	NA
20,8624	0	PR	CR/PR	IC2+	TC0	excluded	4	NA
19,1869	0	PD	SD/PD	IC1	TC0	NA	2	NA
22,8337	0	PD	SD/PD	IC2+	TC0	excluded	1	NA
15,7043	0	PR	CR/PR	IC1	TC0	excluded	59	11,69
21,5852	0	CR	CR/PR	IC1	TC1	excluded	62	10,02
21,4209	0	PR	CR/PR	IC1	TC0	desert	38	6,69

23,1622	0	PR	CR/PR	IC2+	TC2+	inflamed	13	6,00
22,5380	0	PR	CR/PR	IC2+	TC2+	inflamed	NA	5,92
21,3881	0	SD	SD/PD	IC2+	TC0	inflamed	27	5,88
21,4538	0	CR	CR/PR	IC2+	TC2+	inflamed	44	5,82
16,8214	0	CR	CR/PR	IC1	TC2+	excluded	33	5,51
17,2156	0	PR	CR/PR	IC2+	TC0	inflamed	26	4,96
18,1027	0	NE	NA	IC2+	TC2+	excluded	35	4,65
16,5914	0	PR	CR/PR	IC2+	TC0	inflamed	38	4,51
8,9035	0	PD	SD/PD	IC1	TC0	desert	22	4,43
17,1170	0	CR	CR/PR	IC2+	TC1	inflamed	23	4,24
21,2567	0	PD	SD/PD	IC1	TC0	desert	20	4,02
20,7310	0	CR	CR/PR	IC2+	TC0	excluded	35	3,75
20,0411	0	NE	NA	IC2+	TC0	NA	16	3,00
0,1971	0	PD	SD/PD	IC0	TC0	excluded	14	2,92
12,7146	0	PR	CR/PR	IC2+	TC0	excluded	18	2,86
21,5852	0	CR	CR/PR	IC2+	TC0	inflamed	14	2,76
22,0780	0	CR	CR/PR	IC2+	TC0	excluded	15	2,69
19,1211	0	SD	SD/PD	IC1	TC0	inflamed	21	2,39
19,3511	0	CR	CR/PR	IC1	TC1	inflamed	13	2,39
21,0595	0	CR	CR/PR	IC2+	TC0	inflamed	14	2,27
15,4415	0	PD	SD/PD	IC2+	TC1	inflamed	12	2,24
5,5195	0	SD	SD/PD	IC2+	TC0	inflamed	14	2,20
18,8912	0	PR	CR/PR	IC1	TC0	desert	9	2,18
12,8460	0	PR	CR/PR	IC1	TC0	excluded	18	2,12
21,2238	0	PR	CR/PR	IC0	TC0	excluded	8	2,10
19,2854	0	CR	CR/PR	IC2+	TC2+	NA	8	2,08
20,0082	0	PR	CR/PR	IC1	TC0	inflamed	15	1,88
16,8542	0	PR	CR/PR	IC1	TC0	excluded	16	1,86
23,3922	0	PR	CR/PR	IC2+	TC0	excluded	7	1,86
20,6982	0	CR	CR/PR	IC0	TC0	desert	NA	1,82
17,2813	0	PR	CR/PR	IC0	TC0	desert	11	1,82
16,5914	0	PR	CR/PR	IC1	TC0	desert	9	1,80
22,1437	0	CR	CR/PR	IC2+	TC2+	NA	18	1,76
20,5667	0	PR	CR/PR	IC1	TC0	excluded	15	1,73
20,8296	0	CR	CR/PR	IC2+	TC0	excluded	7	1,57
21,1910	0	SD	SD/PD	IC2+	TC0	NA	6	1,57
22,3737	0	PR	CR/PR	IC2+	TC0	inflamed	12	1,53
20,0082	0	PR	CR/PR	IC1	TC0	NA	11	1,51
18,6283	0	NE	NA	IC1	TC0	excluded	10	1,49
17,2813	0	PR	CR/PR	IC2+	TC0	excluded	17	1,41
7,0637	0	PD	SD/PD	IC2+	TC0	excluded	8	1,41
24,1807	0	PR	CR/PR	IC2+	TC0	excluded	5	1,41
20,2710	0	SD	SD/PD	IC2+	TC2+	inflamed	11	1,35
20,6324	0	SD	SD/PD	IC2+	TC0	inflamed	20	1,25
22,7351	0	PD	SD/PD	IC2+	TC0	inflamed	8	1,24
15,8357	0	PD	SD/PD	IC0	TC0	NA	10	1,22
20,5010	0	PR	CR/PR	IC2+	TC0	inflamed	13	1,14
17,2813	0	SD	SD/PD	IC0	TC0	desert	NA	1,10
21,6181	0	PR	CR/PR	IC2+	TC0	excluded	5	1,10
23,2279	0	PR	CR/PR	IC0	TC1	desert	20	1,04
18,5626	0	CR	CR/PR	IC1	TC0	excluded	6	1,04
15,6715	0	SD	SD/PD	IC1	TC0	excluded	7	1,00
23,2608	0	PR	CR/PR	IC0	TC0	NA	6	0,92
17,2813	0	PR	CR/PR	IC0	TC0	NA	19	0,88

20,5667	0	CR	CR/PR	IC0	TC0	desert	14	0,84
18,7269	0	SD	SD/PD	IC2+	TC0	inflamed	8	0,80
23,1294	0	CR	CR/PR	IC2+	TC0	excluded	14	0,78
22,5051	0	PD	SD/PD	IC1	TC0	NA	5	0,78
23,5236	0	CR	CR/PR	IC0	TC0	desert	4	0,76
20,4682	0	SD	SD/PD	IC1	TC0	excluded	12	0,75
21,3224	0	PR	CR/PR	IC1	TC0	excluded	11	0,75
18,8583	0	PR	CR/PR	IC1	TC0	excluded	8	0,75
14,9815	0	PD	SD/PD	IC1	TC0	NA	6	0,75
18,0041	0	NE	NA	IC0	TC0	desert	NA	0,65
21,3881	0	CR	CR/PR	IC2+	TC2+	inflamed	19	0,65
20,7310	0	PR	CR/PR	IC2+	TC0	inflamed	3	0,61
23,8193	0	SD	SD/PD	IC2+	TC2+	inflamed	1	0,61
21,4209	0	PR	CR/PR	IC2+	TC2+	excluded	7	0,57
21,5852	0	SD	SD/PD	IC2+	TC2+	inflamed	6	0,57
2,1027	0	PD	SD/PD	IC0	TC0	desert	5	0,55
19,2854	0	PD	SD/PD	IC1	TC0	inflamed	5	0,49
23,6879	0	SD	SD/PD	IC2+	TC2+	excluded	7	0,35
23,3265	0	SD	SD/PD	IC0	TC0	NA	7	0,35
20,5667	0	PD	SD/PD	IC2+	TC2+	desert	7	0,35
21,8480	0	SD	SD/PD	IC1	TC1	NA	3	0,35
14,7515	0	SD	SD/PD	IC2+	TC0	inflamed	6	0,33
16,4600	0	PR	CR/PR	IC0	TC0	desert	2	0,33
18,7269	0	PR	CR/PR	IC2+	TC2+	NA	1	0,33
14,1273	0	PD	SD/PD	IC1	TC0	NA	4	0,27
19,4168	0	CR	CR/PR	IC1	TC0	inflamed	NA	0,22
14,7515	0	PD	SD/PD	IC1	TC0	inflamed	8	0,22
15,6386	0	SD	SD/PD	IC2+	TC0	excluded	2	0,18

Table_S3 - 87 prognostic TME-related genes from the entire TCGA cohort to establish C1, C2 and C3.

Genes	p-value	HR	Low 95% CI	High 95% CI
ZFP36L1	0,009816	1,275217	1,060336	1,533644
ZCRB1	0,001299	0,634	0,48027	0,836938
WWP1	0,00892	1,353853	1,078851	1,698954
WFDC5	0,00114	1,407553	1,145588	1,729423
WARS	0,008768	0,826133	0,716177	0,952971
VSTM2L	0,002149	1,108654	1,037974	1,184147
VSIG4	0,005528	1,14652	1,040959	1,262786
UBD	0,002261	0,874686	0,802656	0,953179
TSC22D1	0,00317	1,308749	1,094556	1,564857
TRPM2	0,006515	1,268613	1,068788	1,505798
TREM1	0,002822	1,280427	1,08868	1,505947
TMEFF2	0,000736	2,120496	1,370589	3,280709
TCF15	0,009038	1,215116	1,04976	1,406519
SULT4A1	0,004336	1,261211	1,075321	1,479235
STAT5A	0,007757	1,257316	1,062279	1,488162
STAB1	0,006356	1,17721	1,047041	1,323561
SPOCK2	0,009587	1,107048	1,025059	1,195595
SNRPD1	0,000306	0,666996	0,535365	0,830991
SNRPA1	0,003959	0,729027	0,588004	0,903872
SLC7A11	0,008216	0,83187	0,725725	0,953539
SEM1	0,004329	0,691553	0,536776	0,890959
RNF175	5,80E-05	1,761501	1,33666	2,321372
PTGER2	0,006969	1,146558	1,038133	1,266308
PLXNA1	0,006948	1,232299	1,058881	1,434118
PLEKHO2	0,004758	1,295369	1,082328	1,550344
PLEKHF1	0,000717	1,239916	1,094671	1,404432
PLA2G2D	0,005749	0,813662	0,702892	0,941889
PJA2	0,004326	1,389892	1,108583	1,742586
PIR	0,007009	0,830028	0,724908	0,950392
PIEZO1	0,001605	1,206416	1,073667	1,355577
PI3	8,57E-05	1,128353	1,062369	1,198436
PDP1	0,00021	1,432001	1,184356	1,731427
PDE7B	0,009219	1,333962	1,073854	1,657073
PDE1C	0,008427	1,687737	1,143395	2,491226
NUCB2	0,003313	0,765489	0,640458	0,914928
NTN1	0,001676	1,183208	1,065348	1,314106
NOL7	0,003262	0,671323	0,51479	0,875453

NLRP12	0,009961	1,806071	1,152092	2,831278
MGAM	0,001293	2,224357	1,366812	3,619932
MAP3K13	0,005841	0,765761	0,633404	0,925774
LYVE1	0,00318	1,184825	1,058558	1,326153
LPIN3	0,002009	1,31219	1,104396	1,559082
LOXL4	0,00668	1,144995	1,038274	1,262685
LILRA2	0,000301	1,459652	1,189025	1,791874
KIF26B	0,004433	1,174554	1,051346	1,3122
ITGB8	0,003137	1,214768	1,067656	1,38215
ISG20	0,003516	0,804984	0,695856	0,931226
HTR2B	0,004766	1,315127	1,087312	1,590674
HPGDS	0,002966	1,293389	1,091509	1,532607
HMGB3	0,00283	0,763928	0,640147	0,911644
HLA-DOB	0,000849	0,803666	0,706826	0,913773
GTF2F2	0,006699	1,449726	1,108409	1,896144
GRIN2D	0,008672	1,167298	1,039956	1,310233
GRB7	0,002071	1,28567	1,095674	1,508613
GPR27	0,00682	0,856643	0,765804	0,958257
GPR179	0,007175	2,914772	1,336325	6,357653
GPR146	0,000991	1,458036	1,164915	1,824913
GMNN	0,003198	0,788396	0,673124	0,923407
FZD3	0,004309	0,800765	0,687468	0,932735
FCGR3B	0,006681	1,291358	1,073504	1,553424
FBXL20	0,007385	1,365131	1,087126	1,714229
ERBB2	0,008688	1,232347	1,054299	1,440463
EMP1	0,000642	1,253894	1,101121	1,427863
ELK3	0,002839	1,287559	1,090652	1,520016
EFNA5	0,007457	1,186394	1,046782	1,344626
DLEU7	0,00377	1,630668	1,171328	2,270141
DAGLB	0,006903	1,398369	1,096405	1,783499
CYBRD1	0,000459	1,250204	1,103398	1,416542
CXCR2	0,000193	1,538891	1,226789	1,930395
CXCL9	0,009507	0,91699	0,858856	0,97906
CXCL13	0,000961	0,872173	0,804161	0,945938
CXCL11	0,003029	0,896799	0,834493	0,963757
COL8A2	0,006974	1,146908	1,038211	1,266986
CLEC5A	0,003437	1,205296	1,063572	1,365904
CHAD	0,002968	1,268197	1,084214	1,4834
CH25H	0,005788	1,224942	1,06055	1,414816
CD40LG	0,004713	0,672974	0,511337	0,885704
CD3G	0,006322	0,769826	0,63804	0,928832

CD38	0,002064	0,78121	0,667652	0,914083
CCDC34	0,003347	0,756882	0,628368	0,911681
CAPN12	0,007681	1,236707	1,057864	1,445786
C5AR1	0,006487	1,188818	1,049616	1,346481
C1orf61	2,13E-05	8,622169	3,193382	23,27996
BTLA	0,003326	0,639969	0,475065	0,862113
ATP1A3	0,00168	1,144778	1,052171	1,245535
ARHGEF38	0,002073	0,550363	0,376344	0,804847
ANGPT4	0,001039	1,635988	1,219074	2,195482

Table S4. 92 prognostic TME-related genes from TCGA training cohort to establish the RS.

Genes	*p*-value	HR	Low 95% CI	High 95% CI
ZNF747	0,0079	1,23E-06	5,31E-11	0,0284
ZNF320	0,0031	1,10E-05	5,63E-09	0,0213
ZNF239	0,0044	3,16E-06	5,15E-10	0,0194
ZNF165	0,0037	3,76E-06	8,14E-10	0,0173
ZFP36L1	0,0075	1,7035443	1,15259	2,5179
ZBP1	0,0092	7,71E-05	6,23E-08	0,0956
XRCC3	0,005	9,23E-06	2,81E-09	0,0303
XRCC2	0,0054	1,59E-05	6,60E-09	0,0384
WDR91	0,009	0,0003782	1,03E-06	0,1396
UQCRFS1	0,0008	1,9779533	1,3262939	2,9498
TRIM37	0,0073	1,72E-05	5,63E-09	0,0523
TRIM36	0,0082	6,10E-05	4,58E-08	0,0813
TRIM24	0,0072	1,74E-05	5,91E-09	0,0512
THBS1	0,0098	3,2539385	1,3286742	7,9689
TECPR2	0,0074	6,23E-06	9,70E-10	0,04
TBCD	0,0094	1,76E-05	4,52E-09	0,0684
SNRPA1	0,0046	0,0001518	3,47E-07	0,0664
SMAD4	0,0048	4,47E-06	8,60E-10	0,0232
SKA1	0,0079	3,85E-05	2,13E-08	0,0696
SEMA4A	0,0099	0,0001367	1,58E-07	0,1181
SEM1	0,0065	4,84E-05	3,80E-08	0,0618
SELL	0,0012	8,68E-06	7,55E-09	0,01
SACM1L	0,0077	1,79E-06	1,05E-10	0,0304
RPS9	0,0032	1,3669536	1,1103595	1,6828
RPL21	0,0018	1,2735537	1,0942703	1,4822
RAD54L	0,0045	1,89E-05	1,04E-08	0,0345
PRR11	0,0084	9,37E-05	9,50E-08	0,0923
PPP4R3A	0,0023	2,09E-05	2,05E-08	0,0214
POLE2	0,005	1,97E-05	1,02E-08	0,038
POLA2	0,0088	1,81E-05	5,08E-09	0,0642
POC1B	0,0056	5,91E-06	1,19E-09	0,0293
PLEKHF1	0,0007	6,0807591	2,1340738	17,326
PKM	0,0018	1,3625737	1,1214895	1,6555
PIR	0,0026	6,56E-05	1,23E-07	0,035
PI3	0,0007	1,3113772	1,1208382	1,5343
OLFML3	0,0098	5,7569285	1,5259864	21,719
OFD1	0,0082	3,88E-05	2,09E-08	0,0723
NUCB2	0,0062	0,000144	2,57E-07	0,0807
NFATC2IP	0,0034	4,15E-06	1,04E-09	0,0165

NDC80	0,0034	1,40E-05	7,98E-09	0,0245
NCAPG2	0,0058	5,75E-06	1,09E-09	0,0303
MICB	0,0007	2,84E-05	6,73E-08	0,012
MFAP4	0,0008	1,9370878	1,3146197	2,8543
ME1	0,0031	4,20E-06	1,15E-09	0,0153
MAP3K13	0,0022	1,69E-05	1,48E-08	0,0192
MAL	0,0048	1,4112129	1,110634	1,7931
LRRC45	0,0019	4,93E-06	2,20E-09	0,0111
LPAR2	0,0033	0,0003642	1,85E-06	0,0717
KNTC1	0,0078	3,18E-05	1,55E-08	0,0653
KIF21A	0,0036	1,76E-05	1,11E-08	0,0277
KIF18A	0,0068	8,75E-06	1,90E-09	0,0402
ISG20	0,0065	1,02E-05	2,58E-09	0,04
HPDL	0,0018	2,28E-05	2,75E-08	0,0188
HLA_DOB	0,0016	1,22E-05	1,09E-08	0,0136
HHEX	0,009	2,54E-05	9,08E-09	0,0711
GRB7	0,004	2,4690724	1,3347469	4,5674
GCNT1	0,0065	1,70E-05	6,23E-09	0,0465
GALC	0,0097	1,44E-05	3,06E-09	0,0674
FOXA1	0,005	0,0001552	3,39E-07	0,0711
FANCI	0,0038	4,76E-06	1,17E-09	0,0193
FANCG	0,0051	1,16E-05	4,10E-09	0,0329
FAM43A	0,005	6,7435925	1,7803301	25,544
FAM111B	0,0088	4,78E-05	2,81E-08	0,0813
ESM1	0,01	3,43E-05	1,37E-08	0,0855
ERICH5	0,0021	1,52E-05	1,28E-08	0,0179
ENO2	0,0051	67,35599	3,5430607	1280,5
DONSON	0,0039	9,07E-06	3,42E-09	0,024
DLX6	0,0077	0,0001324	1,87E-07	0,0939
DGLUCY	0,0061	4,62E-05	3,69E-08	0,0578
DCAF10	0,0026	9,41E-07	1,14E-10	0,0078
CYREN	0,0005	4,49E-08	3,27E-12	0,0006
CXCL12	0,0071	8,6411679	1,7954989	41,587
CXCL11	0,0011	0,0036149	0,0001221	0,107
CTPS2	0,0033	1,77E-05	1,20E-08	0,0259
CTAG2	0,004	2,571943	1,3505235	4,898
CPXM1	0,0085	1,427581	1,0950929	1,861
COL26A1	0,0022	4,2221679	1,680812	10,606
CHAF1B	0,001	1,48E-06	5,00E-10	0,0044
CEACAM6	0,0053	6,66E-05	7,73E-08	0,0573
CDK5	0,0059	7,13E-05	8,02E-08	0,0635
CDC5L	0,0058	3,34E-06	4,27E-10	0,0262
CD3E	0,0075	9,15E-05	1,00E-07	0,0836
CD3D	0,0072	5,95E-05	4,95E-08	0,0715
CD27	0,0099	5,23E-05	2,92E-08	0,0939
CD2	0,0062	0,0001614	3,10E-07	0,084
CCL19	0,0048	0,0001313	2,65E-07	0,0651
CCDC80	0,003	55,391572	3,8964135	787,45
CCDC34	0,0062	6,04E-05	5,76E-08	0,0634
ARHGEF39	0,0083	1,91E-05	6,01E-09	0,0607
APCDD1	0,0065	0,0106456	0,0004031	0,2811
ABHD5	0,0007	1,54E-07	1,83E-11	0,0013
ABHD3	0,0073	5,10E-05	3,71E-08	0,0701
